# KPNB1-ATF4 induces BNIP3-dependent mitophagy to drive odontoblastic differentiation in dental pulp stem cells

**DOI:** 10.1186/s11658-024-00664-9

**Published:** 2024-11-27

**Authors:** Zeying Zhang, Di Yang, Xiaoyuan Yan, Qiujing Qiu, Jiajie Guo, Lihong Qiu

**Affiliations:** Department of Endodontics, School and Hospital of Stomatology, Liaoning Provincial Key Laboratory of Oral Diseases, China Medical University, 117 Nanjing North Street, Heping District, Shenyang, Liaoning 110002 People’s Republic of China

**Keywords:** Dental pulp stem cell, Odontoblastic differentiation, Mitophagy, ATF4, BNIP3, KPNB1

## Abstract

**Background:**

Differentiating dental pulp stem cells (DPSCs) into odontoblasts is a critical process for tooth self-repair and dentine‒pulp engineering strategies in the clinic. However, the mechanism underlying the regulation of DPSC odontoblastic differentiation remains largely unknown. Here, we demonstrated that BCL-2 interacting protein 3 (BNIP3)-dependent mitophagy is associated with importin subunit beta-1 (KPNB1)-activating transcription factor 4 (ATF4), which promotes DPSC odontoblastic differentiation.

**Methods:**

The key genes involved in DPSC odontogenic differentiation were identified via bioinformatics. Stable silencing or overexpression of BNIP3 was performed to investigate its impact on DPSC differentiation in vitro (*n* ≥ 3). To explore the role of BNIP3 in vivo, tooth root fragments loaded with the hydrogel-transfected DPSC complex were implanted into nude mice (*n* ≥ 6). Dual-luciferase reporter assays and chromatin immunoprecipitation (ChIP) polymerase chain reaction (PCR) were conducted to explore the binding site of ATF4 to the *BNIP3* promoter (*n* ≥ 3). Mitochondrial function experiments were performed to investigate the impact of ATF4-BNIP3 on mitochondria (*n* ≥ 3). Immunoprecipitation (IP) mass spectrometry (MS) was used to investigate the interaction between ATF4 and its binding protein, KPNB1. Plasmids containing wild-type (WT)/mutant (MUT)-nuclear localization signal (NLS) forms of *ATF4* were constructed to determine the specific amino acid residues recognized by KPNB1 and their effects on DPSC odontoblastic differentiation (*n* ≥ 3).

**Results:**

Compared with those in the control group, the levels of autophagy and mitophagy, especially BNIP3-dependent mitophagy, were greater in the DPSC odontoblastic differentiation group (*P* < 0.05). Genetic silencing or overexpression of BNIP3 demonstrated that BNIP3 expression was positively correlated with the transition of DPSCs into odontoblasts both in vitro and in vivo (*P* < 0.05). ATF4 regulates the expression of BNIP3 by directly binding to approximately −1292 to −1279 bp and approximately −1185 to −1172 bp within the BNIP3 promoter region, which is associated with mitophagy and mitochondrial reactive oxygen species (mtROS) levels (*P* < 0.05). Moreover, ATF4 increased mitophagy, mitochondrial function, and cell differentiation potential via BNIP3 (*P* < 0.05). Mechanistically, KPNB1 is a novel interacting protein of ATF4 that specifically recognizes amino acids (aa) 280–299 within ATF4 to control its translocation into the nucleus and subsequent transcription and differentiation processes (*P* < 0.05).

**Conclusions:**

We reported that the critical role of KPNB1/ATF4/BNIP3 axis-dependent mitophagy could provide new cues for the regeneration of the dental pulp‒dentin complex in DPSCs.

**Graphical Abstract:**

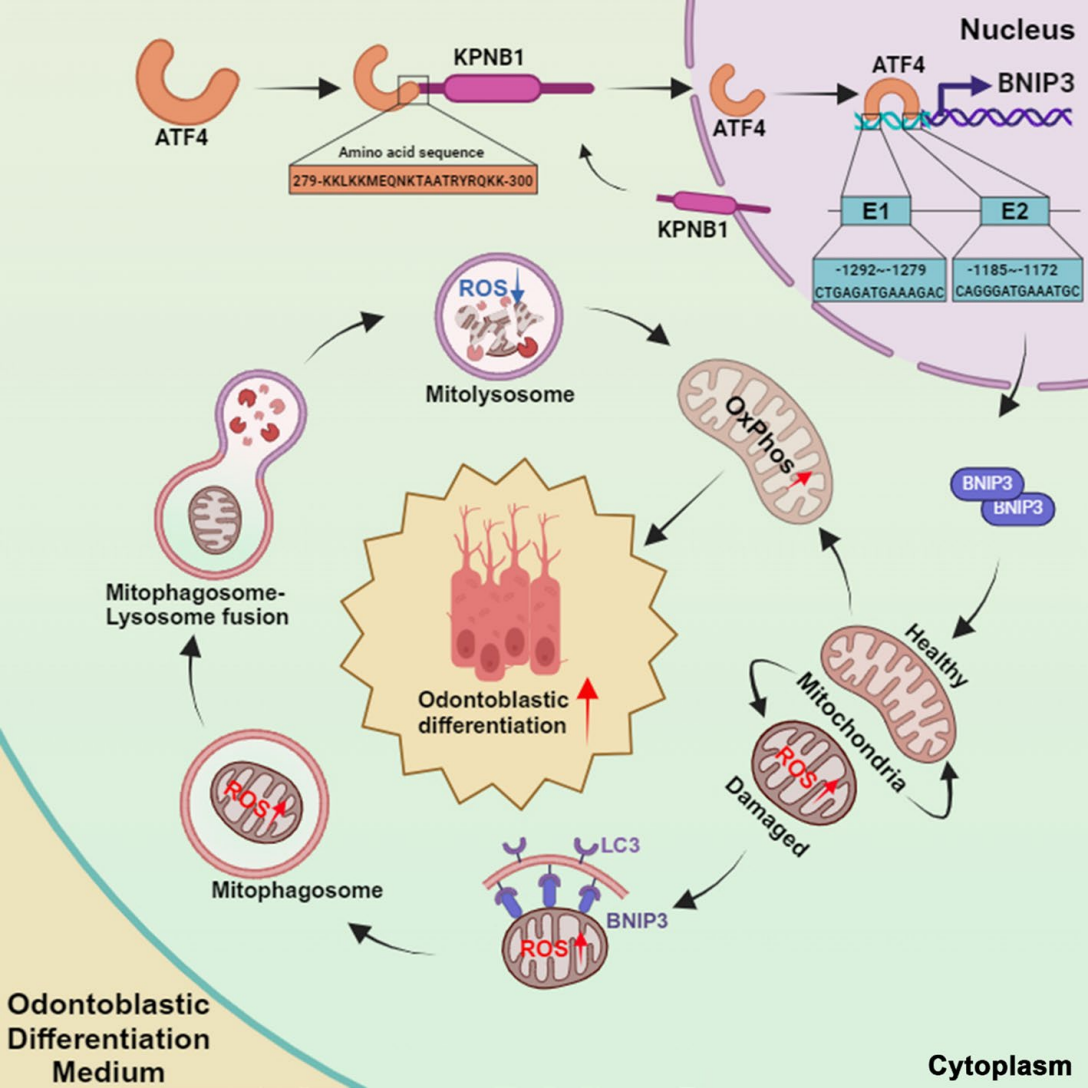

**Supplementary Information:**

The online version contains supplementary material available at 10.1186/s11658-024-00664-9.

## Introduction

Dental pulp stem cells (DPSCs), which reside within dental pulp tissue, possess remarkable proliferative capacity and the potential for multidirectional differentiation [1, 2]. Their abilities to form densely calcified colonies and mineralized nodules have previously been demonstrated [3, 4]. DPSCs can be differentiated by the modulation of growth factors, transcription factors, extracellular matrix proteins and receptor molecules into different cell types, including odontoblasts, osteoblasts, chondrocytes, cardiomyocytes, neuron cells, and adipocytes, both in vitro and in vivo [5–7]. In the realm of regenerative medicine, DPSC-based pulp–dentin complex regeneration is a promising therapeutic approach for regenerating inflamed or necrotic dental pulp tissue, necessitating their directional differentiation into odontoblasts [8–10]. The intricate regulatory mechanisms governing DPSC-directed differentiation and their differentiation capacities constitute a pivotal focus in this field [11]. Despite significant advancements, numerous challenges still impede the widespread clinical application of DPSCs.

Mitophagy is the selective degradation of mitochondria by autophagy. The importance of mitophagy in the osteo-/odontoblastic differentiation process has been proposed [12–14]. During the differentiation process, mitophagy cleans dysfunctional mitochondria, establishes a robust mitochondrial network to supply energy, and serves as the primary source of mineral granules [15, 16]. Hence, mitophagy has emerged as a positive factor in modulating the osteo-/odontoblastic differentiation process of DPSCs. In mammals, PINK1/Parkin is the most classic pathway that mediates mitophagy [17–20] and is best characterized in osteo/odontogenesis promotion by increased mitophagy levels in dental stem cells [21]. However, receptors on the outer mitochondrial membrane that induce mitophagy (PINK1/Parkin independent) are less abundantly investigated in osteo-/odontoblastic differentiation. BCL2-interacting protein 3 (BNIP3) is a novel mitophagy receptor that directly interacts with microtubule-associated protein 1 light chain 3 (LC3) to mediate mitophagy [22]. Recent studies have shown that BNIP3 plays an important role in regulating the differentiation of various stem cells. In *bnip3*^−/−^ mouse myoblasts, increased caspase 3 activity and DNA fragmentation led to increased sensitivity to apoptosis and impaired myogenic differentiation [23]. When *bnip3* deficiency occurs in mouse embryonic stem cells, abnormal mitochondrial accumulation and multiple mitochondrial dysfunctions occur, leading to impaired self-renewal and differentiation potential of stem cells [24]. However, no studies have explored the expression pattern and functional role of BNIP3, as well as the intricate upstream regulatory mechanisms governing mitophagy in odontoblast differentiation.

Activating transcription factor 4 (ATF4), a member of the ATF/CREB family, can act as a transcription factor to activate downstream target gene transcription in an independent or dimeric form [25, 26]. ATF4 can directly regulate not only the transcription of osteogenic differentiation-related marker genes [25, 27] but also long non-coding RNA (lncRNA) to affect histone methylation enzymes, thereby promoting DPSCs odontoblastic differentiation [28]. These findings indicate that the transcriptional regulation of ATF4 is extensive and multidirectional in certain biological phenomena. Recent studies have shown a positive correlation between ATF4 and mitophagy, but whether ATF4 directly affects the transcription of mitophagy-related functional proteins such as BNIP3 has not been elucidated [29, 30]. Moreover, whether the transcription factor ATF4, which is a transcription factor that shuttles between the cytoplasm and the nucleus, is regulated by nuclear transport proteins warrants investigation.

In this study, we present novel insights into the regulation of osteogenic differentiation through BNIP3-dependent mitophagy both in vitro and in vivo. We demonstrated that ATF4 directly governs this process at the transcriptional level. Mechanistically, importin subunit beta-1 (KPNB1) plays a crucial role in facilitating ATF4 nuclear translocation via its nuclear localization signal (NLS), subsequently activating the BNIP3 pathway and promoting odontoblastic differentiation. These findings highlight the mechanism underlying DPSCs odontoblastic differentiation, offer potential targets for achieving pulp-dentin complex regeneration, and provide more tissue engineering strategies involving dental pulp and dentin.

## Materials and methods

### Isolation and characterization of DPSCs

Healthy and intact premolars for orthodontic therapy from young adults aged 18 to 25 were employed to isolate DPSCs under approved guidelines set by the Ethics Committee of the School of Stomatology, China Medical University, Shenyang, China (G2018009). The donors provided written informed consent, granting permission to use their extracted dental tissues for experimental purposes. In brief, the extracted teeth were exposed to phosphate-buffered saline (PBS) supplemented with 2% penicillin (200 U/mL) and streptomycin (0.2 mg/mL) (PS, HyClone, SV30010, USA). Subsequently, pulp tissue fragments were obtained by mechanically disrupting the pulp cavity structure and further treated with 4 mg/mL type II neutral proteinase (Roche, 04942078001, Switzerland) and 3 mg/mL type I collagenase (Invitrogen, 17100017, USA). DPSCs were cultured in α-MEM medium (Gibco, C12571500BT, USA) supplemented with 20% fetal bovine serum (FBS, Clark, FB15015, USA) and 1% PS (penicillin: 100 U/mL and streptomycin: 0.1 mg/mL) until the primary cells migrated from the tissue fragments. Cells in passages three to six were used in subsequent experiments. The stemness capabilities of DPSCs were confirmed through flow cytometric analysis. Furthermore, their diverse differentiation potential was validated using alizarin red S (ARS) staining (Sigma, A5533, USA), oil red O staining (Beyotime, C0158S, China), and alcian blue chondrocyte staining (Cyagen, ALCB-10001, China) after induction 21 days as previously described [31, 32].

### Cell treatment and transfection

DPSCs were cultured in α-MEM supplemented with 10% FBS and 1% PS. When the cell confluence reached approximately 40–50%, transfection was performed following the manufacturer's instructions (Genechem, HiTransG, China). Briefly, DPSCs were transfected with lentiviruses at a multiplicity of infection (MOI) of 25, and the culture medium was replaced after 12 h. To establish stable cell strains, DPSCs were exposed to a culture medium containing 2 μg/mL puromycin for 48 h. The surviving DPSCs were further cultured and utilized in subsequent experiments.

To assess autophagic flux in DPSCs during odontoblastic differentiation, 5 μM rapamycin (Rapa, APExBIO, A8167, USA), or 50 nM bafilomycin A1 (Baf A1, APExBIO, A8627, USA) was added to the odontoblastic medium (OM). DPSCs were cultured following the cycle of “2-day treatment with autophagy inducers or inhibitors 3-day culture without autophagy modifiers”.

To achieve the stable overexpression of ATF4 or BNIP3, as well as the knockdown of BNIP3, lentiviruses carrying oe-ATF4 (Ubi-MCS-3FLAG-SV40-EGFP-IRES-puromycin), oe-BNIP3 (Ubi-MCS-3FLAG-SV40-puromycin), kd-BNIP3 (hU6-MCS-CMV-Puromycin), and their respective negative controls were procured from Genechem, China. The ATF4-WT vector/ATF4-mutant vector, siBNIP3, or shKPNB1 with its negative control respectively were transfected using the jetPRIME transfection reagent (Polyplus, 101,000,046, France). The target sequences of BNIP3 small interference were (RIBOBIO, China):

1#-ACACGAGGTCATGAAGAA; 2#-GTTCCAGCCTCGGTTCTA; 3#-GAACTGCACTTCAGCATA.

For the evaluation of mitophagy, DPSCs were co-transfected with adenovirus Ad-GFP-LC3 and HBAD-Mito-dsRed (Hanbio, China). Mitophagy was visualized and recorded using a laser confocal microscope.

### Alkaline phosphatase (ALP) and ARS staining

To initiate odontoblastic differentiation, DPSCs were cultured in DMEM (Gibco, C11995500BT, USA) supplemented with 10% FBS, 1% PS, 50 μg/mL L-ascorbic acid (Sigma, A4403, USA), 10 mM β-glycerophosphate (Sigma, G9422, USA), and 10 nM dexamethasone (Sigma, D4902, USA). The induction medium was replaced every three days. After 7 days of induction, the cells were fixed with 4% paraformaldehyde (PFA) and stained using the BCIP/NBT alkaline phosphatase kit (Beyotime, C3206, China).

After 21 days of induction, the cells were fixed with 4% PFA and stained using a 0.5% ARS solution (pH = 6.5) prepared by dissolving ARS powder in ddH_2_O. Subsequently, the optical density (OD) value of the stained cells was measured by dissolving them in 10% cetylpyridinium chloride (Aladdin, H108697, China) at 562 nm.

### Immunofluorescence staining

For immunofluorescence staining, the cells were fixed with 4% PFA and permeabilized with 0.5% Triton X-100. Then, the cells were blocked with 1% BSA for 30 min at room temperature. The primary antibodies anti-ATF4 (1:100, CST, 11815, USA) and anti-KPNB1 (1:200, Abcam, ab2811, UK) were incubated with the cells overnight at 4 °C. The goat-anti-mouse or goat-anti-rabbit secondary antibodies (1:500, Proteintech, SA00013-3 or SA00013-2, China) were added and incubated for 2 h at 37 °C. The images were captured using an inverted microscope (Leica, Japan).

### Bioinformatics analysis and quantitative real-time polymerase chain reaction (RT‒qPCR)

RNA-seq data (GSE138179) of DPSCs and DPSCs undergoing odontoblastic differentiation were retrieved from the GEO database. Differentially expressed gene (DEG) analysis was performed using the “edgeR” package with the following criteria: |logFC|> 0.58 and *P* < 0.05. The results are presented in the form of volcano plots and were visualized using the “ggplot” package. Gene Ontology (GO) analysis for the identified pathways enriched in DEGs was performed using the “clusterProfiler” package.

Total RNA was extracted and subjected to reverse transcription following the protocols provided by the reverse kit (Takara, RR047A, Japan). Real-time quantitative polymerase chain reaction (RT‒qPCR) was carried out utilizing the SYBR Green kit (Qiagen, 208,504, Germany) and detected using the ABI 7500 system (Applied Biosystems, USA). Supplementary File 1 contains the list of primer sequences utilized in the experiments. The relative mRNA levels of each sample were calculated using the 2^(−ΔΔCt)^ method, with GAPDH expression as the normalization control.

### Subcellular fractionation and western blots

Nuclear and cytoplasmic proteins of DPSCs were extracted following the instructions provided by the kit from Thermo Fisher, AM1921, USA. For the isolation of mitochondrial proteins, DPSCs mitochondrial protein extraction was conducted according to the protocol supplied by the kit from Beyotime, C3601, China. Total protein was lysed using radioimmunoprecipitation assay (RIPA) lysis buffer supplemented with 1% PMSF.

Western blotting was carried out to assess the relative expression of the proteins, as previously described [30]. The primary antibodies utilized were as follows: ATF4 (1:1000, CST, 11,815, USA), LC3B (1:1000, Abcam, ab51520, USA), P62 (1:1000, CST, 5114, USA), KPNB1 (1:2000, Abcam, ab2811, USA), FLAG (1:1000, CST, 2368, USA), BNIP3 (1:1000, CST, 44060, USA), DMP1 (1:1000, Signalway, 38779, USA), and DSPP (1:200, Santa, SC-73632, USA). GAPDH (1:10000, Proteintech, HRP-60004, China) served as the cytoplasmic internal reference, Histone3 (1:5000, Affinity, BF9211, China) served as the nuclear internal reference, and COX IV (1:10000, Proteintech, 66110–1-Ig, China) served as the mitochondrial internal reference. Subsequently, the membranes were incubated with the appropriate secondary antibodies (1:10000, Proteintech, SA00001-1 or SA00001-2, China). Immunoreactive protein bands were visualized using enhanced chemiluminescence (Tannon, 180-5001, China), and band quantification was conducted within the linear range. The relative expression of the proteins was measured using ImageJ software.

### MitoSOX staining and flow cytometry

The levels of mitochondrial reactive oxygen species (mtROS) in DPSCs were assessed utilizing MitoSOX (Yeasen, 40778ES50, China). DPSCs were cultured in a medium containing 3 μM MitoSOX and incubated at 37 °C in the dark for 30 min. Subsequently, the cells were washed twice with PBS, and the relative fluorescence intensity of MitoSOX was measured using an inverted microscope and a flow cytometer.

### Chromatin immunoprecipitation PCR (ChIP‒PCR) assays

ChIP assays were conducted using the ChIP assay kit from Beyotime, P2078, China. After inducing DPSCs towards odontoblastic differentiation for 3 days, the cells were cross-linked with 1% PFA to a final concentration. Subsequently, they were lysed with lysis buffer and sonicated on ice, with each sonication cycle lasting 10 s and repeated ten times. The input group was stored at −80 °C, while the immunoprecipitation (IP) of sonicated chromatin solutions was carried out by incubating with anti-ATF4 (2 μg, CST, 11815, USA) or IgG (2 μg, Beyotime, A7016, China) antibodies overnight at 4 °C. Protein A + G agarose beads were then added, followed by sequential washes with low salt buffer, high salt buffer, LiCl buffer, and TE buffer. The purified DNA was resuspended in TE buffer, and the protein‒DNA complexes were eluted with the reversal of cross-linking. DNA samples were purified and subsequently amplified by PCR (Takara, RR001A, Japan). A 2% agarose gel electrophoresis was performed, and the results were visualized through ultraviolet fluoroscopy. The primer sequences for the ATF4 binding sites of BNIP3 are provided in Supplementary File 2.

### Luciferase reporter assay

The luciferase reporter assay was performed following the protocol provided in the dual-luciferase reporter gene assay kit from Yeasen (11402ES). In brief, HEK 293 T cells were cotransfected with various plasmids, including ATF4-vector, wild-type (WT) vector of the BNIP3 promoter, mutant 1 (MUT1) vector of the BNIP3 promoter, mutant 2 (MUT2) vector of the BNIP3 promoter, mutant 1 and 2 (MUT1 and 2) vector of the BNIP3 promoter, and negative control plasmids. Additionally, the pGL-HRE-AdML reporter plasmid and the pRL-tk vector served as the internal normalization controls (Taihe Biotechnology, China).

### Coimmunoprecipitation (Co-IP) assay and mass spectrometry assay

For Co-IP in DPSCs, the Pierce Crosslink Magnetic IP/Co-IP Kit (Thermo Fisher, 88805, USA) was employed, following the manufacturer's instructions. In brief, primary antibodies or isotype control IgG were covalently cross-linked to 25 μL of protein A/G magnetic beads. Proteins were extracted using IP lysis/wash buffer supplemented with PMSF and a protease and phosphatase inhibitor cocktail. A portion of the sample was saved as an input for later comparison. An equal amount (1 mg) of each protein extract was incubated with protein A/G magnetic beads cross-linked with primary antibodies or isotype control IgG overnight at 4 °C. The beads were then washed with elution buffer and neutralization buffer to obtain the protein. The samples were subsequently analyzed by western blotting, as described in the section Subcellular fractionation and western blots.

For the liquid chromatography tandem mass spectrometry (LC–MS/MS) assay, Co-IP was performed using the Pierce Classic Magnetic IP/Co-IP kit (Thermo Fisher, 88804, USA) under the manufacturer’s instructions. In this case, 1 mg of protein lysate was incubated with 10 mg of primary antibodies or isotype control IgG overnight at 4 °C. Pierce Protein A/G Magnetic Beads were washed three times using Pierce IP Lysis/Wash Buffer for LC–MS/MS analysis. LC–MS/MS sequencing was conducted by BIOPROFILE (Shanghai, China).

### Oxygen consumption rate (OCR)

The OCR was assessed by the Seahorse Cell Mito Stress Test in real-time using the 24-well Extracellular Flux Analyser XF-24 (Agilent, 103,015, USA). Briefly, DPSCs were seeded in each well of the microplate, and the transfected cells were incubated overnight in 200 µl of medium under different conditions. The microplates were then equilibrated without CO_2_ for 1 h before measurement. Subsequently, the ATPase inhibitor oligomycin (Oligo, 1 µM), the uncoupling reagent carbonyl cyanide-*P*-trifluoromethoxyphenylhydrazone (FCCP, 1 µM), and inhibitors of the electron transport chain rotenone/antimycin (R/A, 2 µM) were sequentially injected during real-time measurements of the OCR, whereas respiratory parameters were measured after each injection. The following respiratory parametes were assessed: basal cellular respiration: difference between the last rate measurement before the oligo injection and non-mitochondrial respiration (minimum rate measurement after the R/A injection); maximal respiration: differences between the maximum rate measurement after FCCP injection and the non-mitochondrial respiration; spare respiratory capacity: differences between the maximum respiration and basal respiration; ATP production: difference between the final rate measurement before the oligo injection and the minimum rate measurement after the oligo injection; and proton leak: difference between minimum rate measurement after oligomycin injection and nonmitochondrial respiration.

### Animal experiments

To investigate the impact of BNIP3 on DPSCs odontoblastic differentiation in vivo, we used a tooth fragment in vivo mouse model. All animal procedures were approved and conducted following the guidelines of the Institutional Animal Care Committee of China Medical University (CMU2022014) for the odontoblastic differentiation animal model. Single-root human teeth were prepared as follows: The tooth roots were divided into short segments (~ 5 mm), followed by cleaned and shaped using rotary instruments. All fragments were treated with 17% ethylenediaminetetraacetic acid (EDTA) for 10 min and 5.25% NaClO for 15 min to eliminate the organic component, debris, and microorganisms. All segments were incubated in a culture medium at 37 °C before use. DPSCs transfected with non-targeting control (nc-oe-BNIP3), BNIP3 overexpression (oe-BNIP3), nontargeting control (nc-kd-BNIP3), or BNIP3 knockdown (kd-BNIP3) were encapsulated in 6% EFL-GM-PR hydrogel (EFL, EFL-GM-PR, China) and injected into the root canals. After overnight incubation with culture medium, four root segments (nc-oe-BNIP3; oe-BNIP3; nc-kd-BNIP3; kd-BNIP3) were transplanted subcutaneously into each dorsum of the male nude mouse (a total of 6 nude BALB/c mice, 8 weeks old, weighing 18–22 g, male). After 8 weeks, the samples were retrieved, fixed with 4% PFA for 24 h, and demineralized with EDTA for 8 weeks. Haematoxylin–eosin (HE), immunofluorescence (IF), and immunohistochemistry (IHC) analyses were performed on 4-μm thick paraffin sections. HE staining assay was undergone according to the manufacturer's instructions (Solarbio, G1120, China). IHC staining was performed using an IHC kit (Gene Tech, RR001A, China) procedure to detect DSPP and DMP1 in paraffin-embedded tooth fragment sections. The primary antibodies were DSPP (1:100, Santa, SC-73632, USA) and DMP1 (1:200, Signal way, 38779, USA) followed by secondary antibodies, and counterstained with hematoxylin. Images were acquired and quantified immunohistochemistry was determined by the relative intensity of the positive area using Image-J. IF staining was similarly to IHC, the section was incubated with primary antibody: BNIP3 (1:100, CST, 44060, USA), ATF4 (1:100, CST, 11815, USA), DSPP (1:100, Santa, SC-73632, USA), or DMP1 (1:250, Santa, SC-81249, USA) followed by fluorescent secondary antibody, and DAPI-labeled nucleus (Beyotime, P0131, China).

### Statistical analysis

Statistical analyses were conducted using R language (version 3.5) and GraphPad Prism 7 software. The data are presented as the means ± standard deviation (SD). Two-group comparisons were conducted using unpaired two-tailed Student’s *t*-tests. Multiple-group comparisons were analysed using one-way ANOVA or two-way ANOVA, followed by Dunnett's multiple comparisons, Tukey’s multiple comparisons tests, or Šidák’s multiple comparisons tests. Nonparametric tests were applied if the data showed a skewed distribution. Each assay was independently performed at least three times. A significance level of *P* < 0.05 was considered statistically significant.

## Results

### Autophagy promoted odontoblastic differentiation of DPSCs

**Fig. 1:**
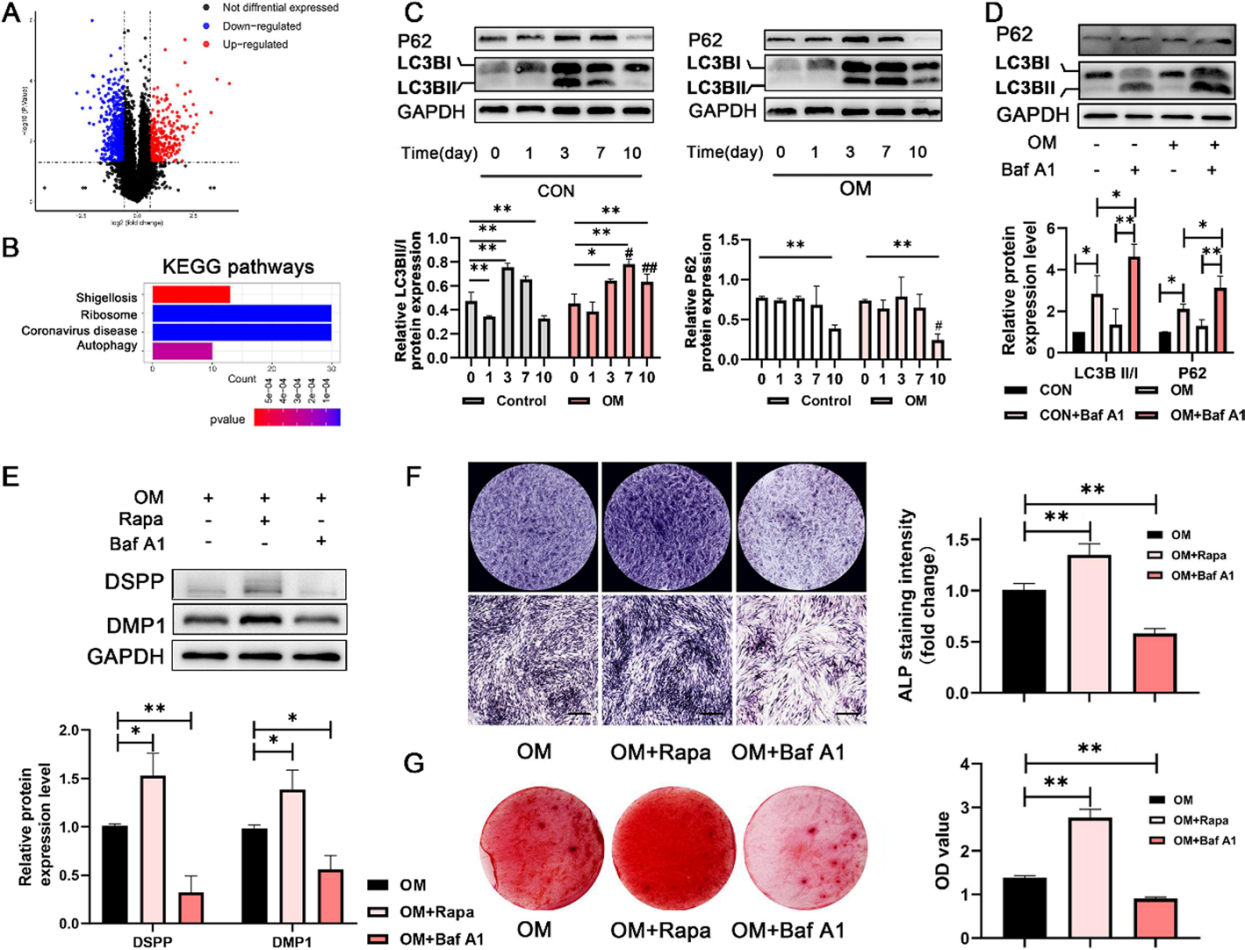
Odontoblastic differentiation induces autophagy and autophagy-related genes. **A** Bioinformatic analysis revealed key DEGs induced by odontoblastic OM at the transcriptional level. **B** The KEGG term plot revealed that “autophagy” pathways were significantly enriched for DEGs in DPSCs cultured with OM compared with those cultured with control medium. **C** Autophagic protein markers were detected in DPSCs cultured with control medium or OM via western blot analysis. The lower panel shows the quantitative representation of the western blot band intensities. **D** Autophagic flux was detected by examining the accumulation of LC3BII/I and the decomposition of P62 after Baf A1 periodic treatment (7 days). The lower panel shows the quantitative representation of the western blot band intensities. ^#^*P* < 0.05 and ^##^*P* < 0.01 indicate significant differences between the control and the OM groups at the same timepoint. **E** The expression of odontoblastic marker proteins was detected to evaluate the role of autophagy in DPSC differentiation (7 days). The lower panel shows the quantitative representation of western blot band intensities. **F** ALP staining after DPSCs were periodically cultured in OM for 7 days with Rapa or Baf A1. Scale bar, 500 μm. The right panel shows the semiquantification of ALP staining intensity. **G** ARS staining after DPSCs were periodically cultured in OM for 21 days with Rapa or Baf A1. The right panel shows the quantification of stained calcium deposits at 562 nm in DPSCs The data are presented as the means ± standard deviation (SD). *N* ≥ 3; *P < 0.05 and **P < 0.01 indicate significant differences between the indicated columns

Isolated DPSCs were characterized to confirm their mesenchymal stem cell nature (Fig. S1). After DPSCs were subjected to OM differentiation for 14 days, RNA sequencing (RNA-seq) analysis of the transcriptome was conducted. In total, 281 upregulated DEGs and 609 downregulated DEGs were involved in osteogenic differentiation (Fig. 1A). These DEGs were enriched in functional categories related to “autophagy” (Fig. 1B). The protein expression of the autophagy markers microtubule-associated protein 1 light chain 3 beta (LC3B) and ubiquitin-binding protein p62 (P62), which serve as negative indicators of autophagic activity, was subsequently evaluated to verify the pathway analysis. The ratio of LC3B II/I in both groups peaked on the third day. The LC3B II/I ratio in the control group subsequently tended to decrease, whereas the ratio in the OM group continued to increase. Compared with that in the control group, the LC3B II/I ratio in the differentiation group significantly increased during the middle and late stages of cell culture (7 and 10 days). Moreover, the expression of P62 in the differentiation group was markedly reduced at the late stage of cell culture (10 days), suggesting that autophagy levels were significantly greater in DPSCs cultured under odontoblastic-inducing conditions with OM than in those cultured in normal medium, which is consistent with the results of the bioinformatics analysis (Fig. 1C).

Bafilomycin A1 (Baf A1) can block the fusion of autophagosomes and lysosomes, allowing degraded substrates to accumulate within the cell, which indirectly reflects the cellular autophagic flux. The results revealed that the application of Baf A1 led to increased P62 and LC3B II/I accumulation in the OM, indicating increased autophagic flux during the differentiation process of DPSCs (Fig. 1D). Further experiments confirmed the impact of autophagy on odontoblastic differentiation of DPSCs. The expression of the odontoblastic differentiation markers DSPP and DMP1 (Fig. 1E), ALP activity (Fig. 1F), and alizarin activity (Fig. 1G) were increased by periodic supplementation with autophagy agonists, specifically rapamycin (Rapa), in the OM but inhibited by Baf A1. These results validate the vital role of autophagy in the induction of DPSC differentiation into odontoblast-like cells.

### BNIP3-dependent mitophagy increased during DPSC odontoblastic differentiation

**Fig. 2:**
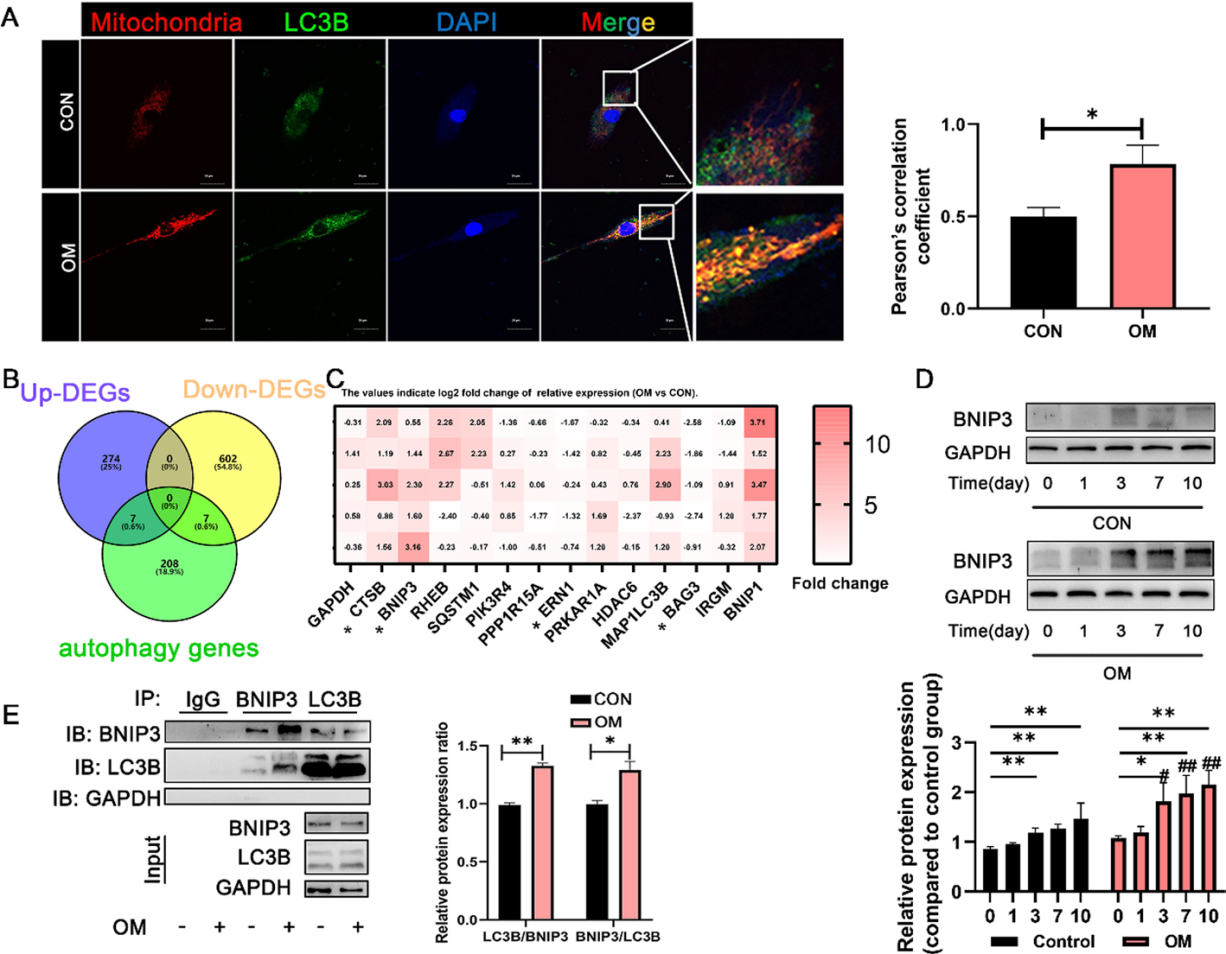
BNIP3-dependent mitophagy increased during DPSC odontoblastic differentiation. **A** Mitochondria‒autolysosome fusion in DPSC differentiation into odontoblasts (versus undifferentiated DPSCs) was evaluated during the early stage of osteogenic induction (3 days) by immunofluorescence (green: LC3-EGFP; red: mito-dsRED; and blue: nucleus). Scale bar, 20 μm. **B** A Venn diagram indicates the autophagy-related DEGs in DPSCs differentiated into odontoblasts. **C** DEGs related to autophagy between DPSCs incubated in control medium and those incubated in OM by RT‒qPCR were verified. The log_2_ fold change values for autophagy-related DEGs are displayed on the heatmap. **D** BNIP3 protein was assessed in DPSCs cultured in normal medium or differentiation medium. The lower panel shows the results of the relative quantitative analysis of BNIP3. ^#^*P* < 0.05 and ^##^*P* < 0.01 indicate significant differences between the control and OM groups at the same time point. **E** The binding of BNIP3 to LC3B in DPSCs differentiated into odontoblasts (3 days) were detected via coimmunoprecipitation assays. The right panel shows the results of the relative quantitative analysis. The data are presented as the means ± SD. *N* ≥ 3; *P < 0.05 and **P < 0.01 indicate significant differences between the indicated columns

Given the pivotal role of mitophagy in the differentiation of DPSCs, the level of mitophagy in DPSCs treated with either growth medium or odontogenic differentiation medium for 3 days was assessed via fluorescence staining. The results indicated that DPSCs treated with OM presented higher levels of mitochondria and autophagosomes than those treated with CON did (Fig. 2A). Moreover, the colocalization signal of mitophagy was significantly increased, which indicates increased cytoplasmic mitophagy and its crucial role in the differentiation process of DPSCs (Fig. 2A). After the autophagy-related DEGs was analyzed via a Venn diagram, we identified a set of seven upregulated and seven downregulated autophagy-related DEGs (Fig. 2B). RT‒qPCR analysis subsequently confirmed the expression changes of autophagy-related DEGs, including CTSB, ERN1, BAG3, and BNIP3 (Fig. 2C). Western blotting was carried out to examine the expression of the mitophagy-related protein BNIP3. BNIP3 protein levels increased in a time-dependent manner in both groups (0 days, 1 day, 3 days, 7 days, and 10 days), with the odontogenic differentiation induction medium group showing more pronounced expression (Fig. 2D). Further Co-IP revealed greater colocalization of BNIP3 with LC3B, suggesting that BNIP3-dependent mitophagy may play a crucial role in the process of DPSC differentiation (Fig. 2E). These data indicate a positive correlation between BNIP3-dependent mitophagy and the odontoblastic differentiation of DPSCs.

### BNIP3-induced DPSC odontoblastic differentiation in vitro and in vivo

**Fig. 3:**
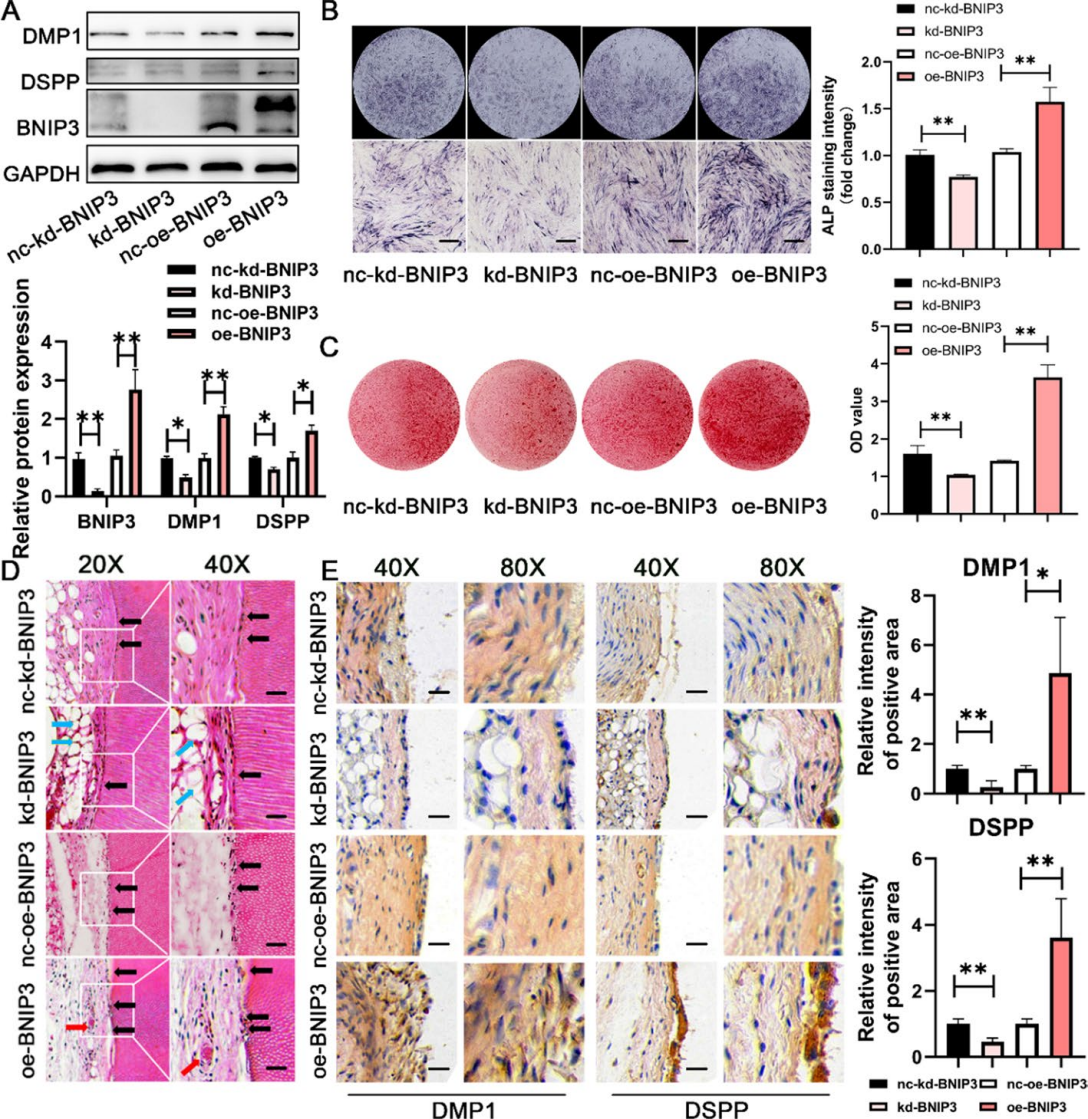
The mitophagy receptor protein BNIP3 is essential for DPSC odontoblastic differentiation. **A** Western blotting was performed to examine the influence of the BNIP3 gene on odontoblastic marker expression (7 days). The lower panel shows the results of the relative quantitative analysis. **B** Odontoblastic differentiation levels were determined via ALP staining (7 days) after stable BNIP3 knockdown or overexpression. Scale bar, 500 μm. The right panel shows the semiquantification of ALP staining intensity. **C** Odontoblastic differentiation levels were determined via ARS staining (21 days) after stable BNIP3 knockdown or overexpression. The right panel shows the quantification of stained calcium deposits at 562 nm in DPSCs. **D**–**E** A gain/loss-of-function study revealed that the mitophagy receptor protein BNIP3 guards DPSCs from differentiating into odontoblasts in vivo via HE staining (**D**) and IHC staining (**E**). Scale bar, 50 μm (40×). Black arrows: pulp-like tissue; blue arrows: vacuolar structure; and red arrows: neovascularization. The right panel shows the semiquantitative intensity of DMP1- and DSPP-positive staining. *N* ≥ 6. The data are shown as the mean ± SD for *N* from 3 to 8; *P < 0.05 and **P < 0.01 indicate significant differences between the indicated columns

To assess the potential impacts of BNIP3 on DPSC differentiation into odontoblasts, we used lentiviruses carrying BNIP3 cDNA (oe-BNIP3) and lentiviruses with BNIP3 interfering RNAi (kd-BNIP3) to overexpress or knock down BNIP3. Compared with the negative control kd-nc-BNIP3 transfection group, the kd-BNIP3 transfection group presented lower levels of DSPP and DMP1 protein expression in DPSCs induced for 7 days (Fig. 3A), reduced ALP activity (Fig. 3B), and showed fewer alizarin red S-stained mineralized nodules (Fig. 3C). Conversely, transfection with oe-BNIP3 tended to promote the above osteogenic indicators.

Additionally, tooth root fragments from DPSCs with silenced or overexpressed BNIP3 were implanted into nude mice to analyze the impact of BNIP3 on DPSC differentiation in the vivo. Similar to the in vitro findings, BNIP3 promoted the odontoblastic differentiation of DPSCs in vivo. As shown by the HE staining results (Fig. 3D), compared with those in the control group, many vacuolar structures (blue arrows) appeared in the root segments of the kd-BNIP3 group, and the newly formed pulp-like tissue was sparse, shallow, and disorderly arranged (black arrows). In contrast, collagen fibers were abundant and highly mineralized in the root segments, and a polar arrangement of odontoblast-like cells was observed on the inner wall of the root canal in the oe-BNIP3 group (black arrow). The cell protrusions penetrated the dentin tubules, and vascular neovascularization containing many bright red blood cells (red arrow) was observed to be scattered, forming a dental pulp dentin complex structure. This positive influence was also observed in the expression levels of odontoblastic marker proteins. As shown in Fig. 3E, BNIP3 knockdown led to decreased DMP1 and DSPP protein expression, whereas BNIP3 overexpression resulted in the opposite result (Fig. 3E). Similarly, the IF staining data supported the above results (Fig. S2). These data indicate that BNIP3 plays an important role in promoting the osteogenic differentiation of DPSCs.

### ATF4 directly engaged in *BNIP3* transcription.

**Fig. 4:**
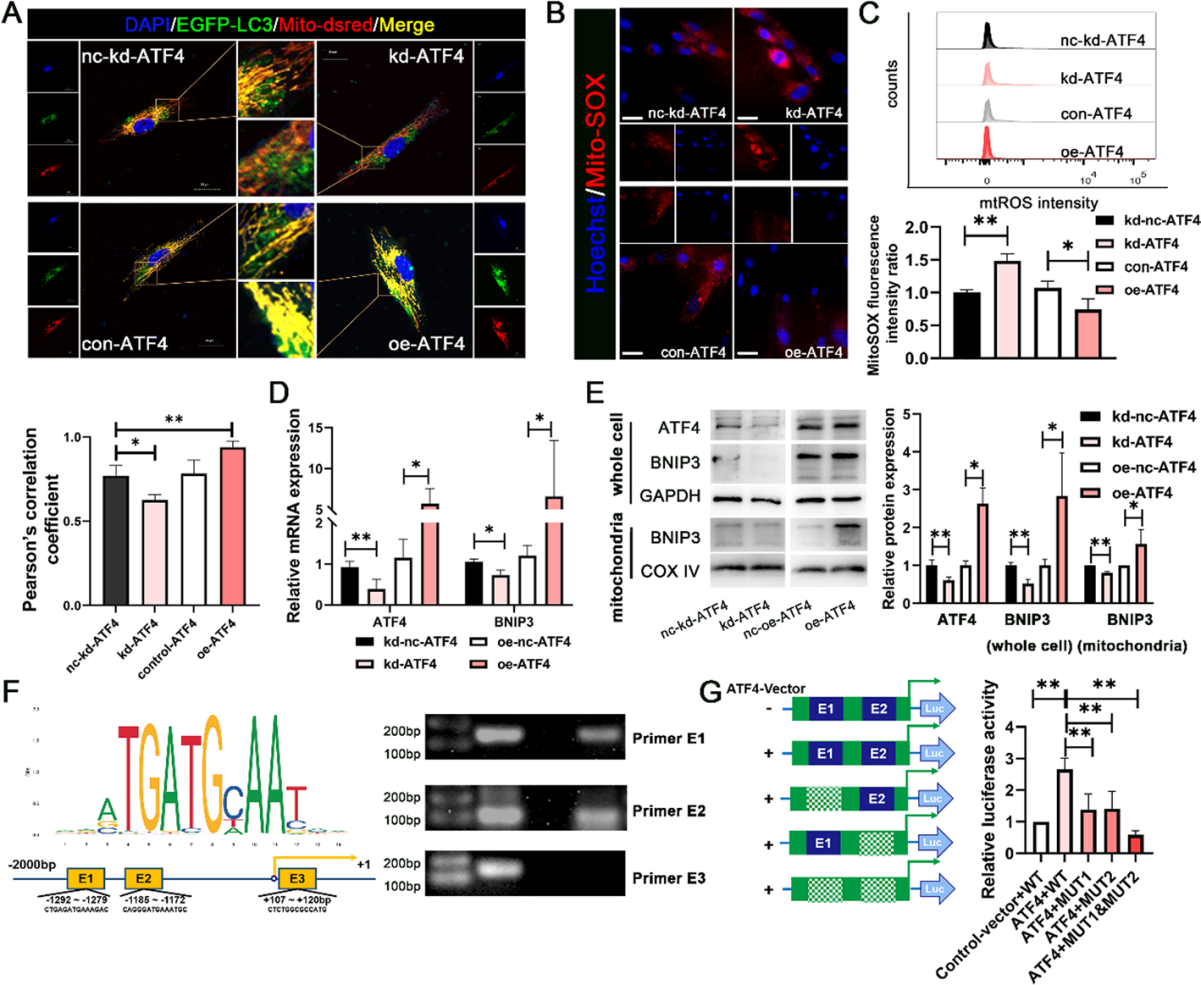
ATF4 induces mitophagy and *BNIP3* transcription by binding its promoter region directly. (**A**)The degree of mitochondria‒autolysosome fusion in DPSCs after ATF4 kd/oe transfection was determined (3 days) via immunofluorescence (green: LC3-EGFP; red: mito-dsRED; and blue: nucleus). Scale bar, 20 μm. **B**,**C** mtROS levels in DPSCs under ATF4 kd/oe transfection conditions were determined via IF (**B**) and flow cytometric analysis (**C**) after incubation with the MitoSOX probe. The lower panel shows the quantification of mtROS levels. **D** RT‒qPCR revealed ATF4 and BNIP3 expression at the transcriptional level. **E** Western blotting revealed ATF4 and BNIP3 expression at the translational level in whole cells and mitochondria. The right panel shows the quantitative representation of the western blot band intensities. **F** Preference binding sequence of the transcription factor ATF4 to target genes from the JARSPAR database. Binding potential between multiple sites in the ATF4 and *BNIP3* promoter regions, which was predicted via the JASPAR website. The right panel shows that the potential binding sites in the ATF4 and *BNIP3* promoter regions were verified (approximately −1292 to −1279 bp and approximately −1185 to −1172 bp) via ChIP‒PCR in DPSCs cultured in OM for 3 days. **G** Schematic of plasmid construction for the dual-luciferase reporter experiment. Dual-luciferase reporter experiments confirmed effective sequences that affect the transcription of the binding site of ATF4 to the *BNIP3* promoter region. The data are shown as the mean ± SD for *N* from 3 to 8; *P < 0.05 and **P < 0.01 indicate significant differences between the indicated columns.

To further clarify the upstream regulatory mechanism of BNIP3, we focused on ATF4, a transcription factor that plays an important regulatory role in mitophagy and osteogenic differentiation. As shown in Fig. 4A, knockdown of ATF4 caused a decrease in intracellular mitophagy signals (indicated by yellow puncta with overlapping red puncta and green puncta), whereas overexpression of ATF4 yielded the opposite results. Mitophagy involves cells selectively eliminating damaged mitochondria to maintain the balance of mitochondrial reactive oxygen species (mtROS). The mtROS staining (Fig. 4B) and flow cytometry results (Fig. 4C) revealed an increase in intracellular mitochondrial superoxide levels upon ATF4 knockdown. These findings strongly suggest that ATF4 could modulate mitophagy and mtROS during the promotion of DPSC odontoblastic differentiation.

When the relationship between ATF4 and BNIP3 was explored, the RT‒qPCR results revealed that the mRNA expression of BNIP3 tended to decrease and then increase with the knockdown and overexpression of ATF4, respectively (Fig. 4D). The western blotting results also confirmed the regulatory effect of ATF4 on BNIP3 expression, both at the whole-cell level and in the mitochondria (Fig. 4E). On the basis of their binding preferences, ATF4 binding motifs were subsequently predicted via the JASPAR website. This result suggested that the potential direct binding sites for ATF4 in the *BNIP3* promoter region were at approximately −1292 to −1279, approximately −1185 to −1172, and approximately + 107 to + 120 bp. Specific primers were designed to target these binding sites, and PCR amplification of the ChIP products confirmed the ability of the anti-ATF4 antibody to pull down DNA sequences at the approximately −1292 to −1279 and approximately −1185 to −1172 bp regions (Fig. 4F). Subsequently, mutant plasmids were constructed to target these two regions, followed by a dual-luciferase reporter assay. The results confirmed that the approximately −1292 to −1279 and approximately −1185 to −1172 regions of the *BNIP3* promoter are active binding sites for ATF4 (Fig. 4G). Furthermore, when both sites were mutated simultaneously, the transcriptional activity of ATF4 on BNIP3 was significantly reduced, indicating that ATF4 likely binds to both sites concurrently to promote *BNIP3* transcription (Fig. 4G). ATF4 regulates the expression of BNIP3 by directly binding to the *BNIP3* transcriptional region.

### BNIP3 inhibition compromised the acceleration of DPSC mitochondrial function caused by ATF4 overexpression

**Fig. 5:**
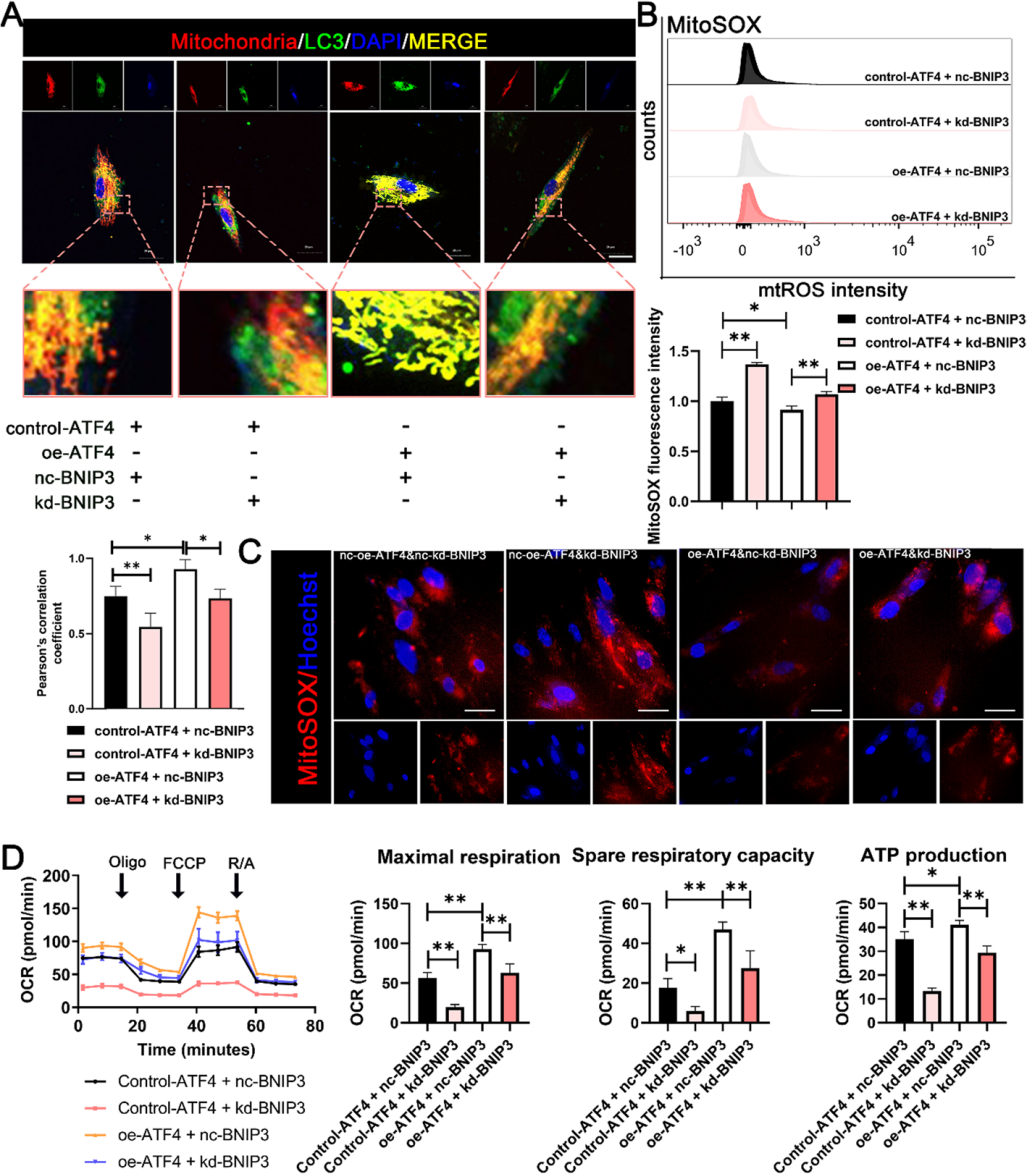
Inhibition of BNIP3 impaired the increased mitochondrial function of DPSCs caused by ATF4 overexpression. **A**. Mitochondria‒autolysosome fusion in DPSCs under various ATF4‒BNIP3 transfection conditions (for 3 days) was evaluated by immunofluorescence (green: LC3‒EGFP; red: mito‒dsRED; and blue: nucleus). Scale bar, 20 μm. **B**-**C**. mtROS levels in DPSCs under various ATF4‒BNIP3 transfection conditions were determined via flow cytometric analysis (**B**) and IF (**C**) after incubation with the MitoSOX probe. **D**. The OCRs of DPSCs transfected with relevant genes were determined via a Seahorse Bioscience XF Analyser. The right panel shows the quantification of maximal respiration, spare cellular respiratory capacity, and ATP production. The data are shown as the mean ± SD for *N* from 3 to 8; *P < 0.05 and **P < 0.01 indicate significant differences between the indicated columns

Having established ATF4 as an upstream effector of BNIP3, we further explored the impact of the ATF4-BNIP3 axis on mitochondrial functions during the differentiation process of DPSCs. Fluorescence colocalization analysis of the mitochondrial–autophagosome complexes revealed that, in DPSCs, the degree of mitophagy colocalization was lower in the rescue group co-transfected ATF4 and siBNIP3 than in the ATF4-overexpressing group (Fig. 5A; Fig. S3). In addition, the reduction in intracellular mtROS caused by ATF4 overexpression during DPSC odontoblastic differentiation was reversed by BNIP3 knockdown (Fig. 5B-C). To provide a comprehensive understanding of the impact of ATF4-BNIP3 on the mitochondrial function of DPSCs during odontoblastic differentiation, the oxygen consumption rate (OCR) of DPSCs was determined via a Seahorse Bioscience XF Analyser. ATF4 overexpression increased maximal respiration, spare respiratory capacity, and ATP production in mitochondria (Fig. 5D). However, upon ATF4 overexpression, BNIP3 knockdown partially suppressed the positive influence of ATF4 on mitochondria. Thus, during the process of odontoblastic differentiation in DPSCs, the enhancement of mitochondrial function and odontoblastic differentiation levels driven by ATF4 primarily depends on BNIP3 mitophagy.

### BNIP3 inhibition compromised the acceleration of DPSC odontoblastic differentiation caused by ATF4 overexpression

**Fig. 6:**
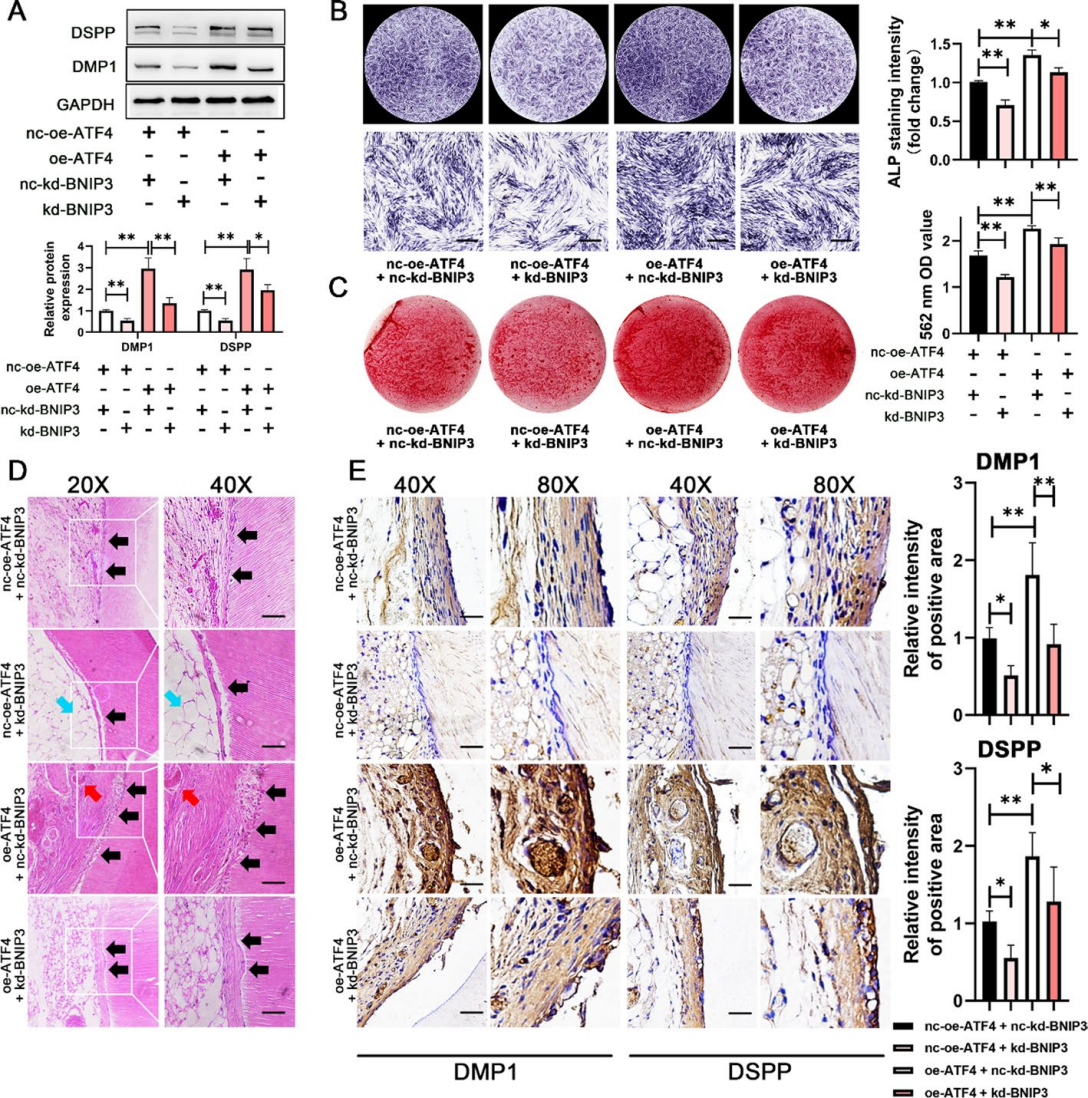
Inhibition of BNIP3 impaired the increased odontoblastic differentiation potential of DPSCs caused by ATF4 overexpression. **A** Western blotting was used to examine the influence of ATF4/BNIP3 on odontoblastic marker expression (after 7 days). The lower panel shows the quantitative representation of the western blot band intensities. **B**. Effects of ATF4/BNIP3 on ALP activity in DPSCs, as determined by ALP staining (7 days). Scale bar, 500 μm. The right panel shows the semiquantification of ALP staining intensity. **C** Effects of ATF4/BNIP3 on calcium deposits in DPSCs, as determined by ARS staining (21 days). The right panel shows the quantification of stained calcium deposits at 562 nm in DPSCs. **D**,**E** ATF4 promoted DPSC differentiation into odontoblasts via BNIP3 in vivo, as shown by HE staining (**D**) and IHC staining (**E**). Scale bar, 50 μm (40×). Black arrows: pulp-like tissue; blue arrows: vacuolar structure; and red arrows: neovascularization. The right panel shows the semiquantitative IHC staining intensity of DMP1- and DSPP-positive staining areas. *N* ≥ 6. The data are shown as the mean ± SD for *N* from 3 to 8; *P < 0.05 and **P < 0.01 indicate significant differences between the indicated columns

Subsequent investigations into odontoblastic differentiation regulated by ATF4-BNIP3 revealed that the ATF4-mediated promotion of odontoblastic differentiation was hindered upon co-transfection with BNIP3 RNAi in vitro and in vivo. DPSCs overexpressing ATF4 presented increased expression of odontoblastic marker proteins (Fig. 6A) and ALP (Fig. 6B) and increased alizarin red S-stained mineralized nodules (Fig. 6C). Moreover, BNIP3-knockdown virus induced a partial deprivation of odontoblastic differentiation in DPSCs overexpressing ATF4 (Fig. 4A-C). This observation underscores the role of BNIP3 as a downstream mediator of ATF4 in facilitating odontoblastic differentiation of DPSCs.

The aforementioned subcutaneous ectopic regeneration in a nude mouse model was used to validate the influence of ATF4/BNIP3 on the odontoblastic differentiation capacity of DPSCs in vivo. HE staining revealed that, compared with the double-negative control group, the oe-ATF4 + nc-BNIP3 group formed a denser layer of odontoblast-like cells accompanied by abundant vascular-like tissue structures. Conversely, the nc-oe-ATF4 + kd-BNIP3 group presented a shallower layer of odontoblast-like cells with adipose-like tissue structures, which was consistent with the in vivo experimental results in which BNIP3 was knocked down (Fig. 3D). Furthermore, compared with that in the oe-ATF4 + nc-kd-BNIP3 group, the formation of odontoblast-like structures in the oe-ATF4 + kd-BNIP3 group was significantly lower, indicating that ATF4 promotes DPSC odontogenic differentiation through BNIP3 (Fig. 6D). IHC staining for the odontoblast-related markers DSPP and DMP1 was conducted to further characterize the newly formed tissue. The results demonstrated that DSPP- and DMP1-immunoreactive cells were distributed mainly on the surface of the newly formed odontoblast-like tissue (Fig. 6E). Compared with the double-negative control group, the oe-ATF4 + nc-kd-BNIP3 group presented higher expression levels of DSPP and DMP1, whereas knockdown of BNIP3 in the oe-ATF4 + kd-BNIP3 group significantly impaired the promoting effect of ATF4 on DSPP and DMP1. Similarly, the IF staining data supported the above results (Fig. S4).

In summary, these data suggest that ATF4 relies on BNIP3 to promote the odontogenic differentiation of DPSCs both in vitro and in vivo.

### KPNB1 is responsible for recognizing the NLS of ATF4 to regulate *BNIP3* transcription and DPSC odontoblastic differentiation

**Fig. 7:**
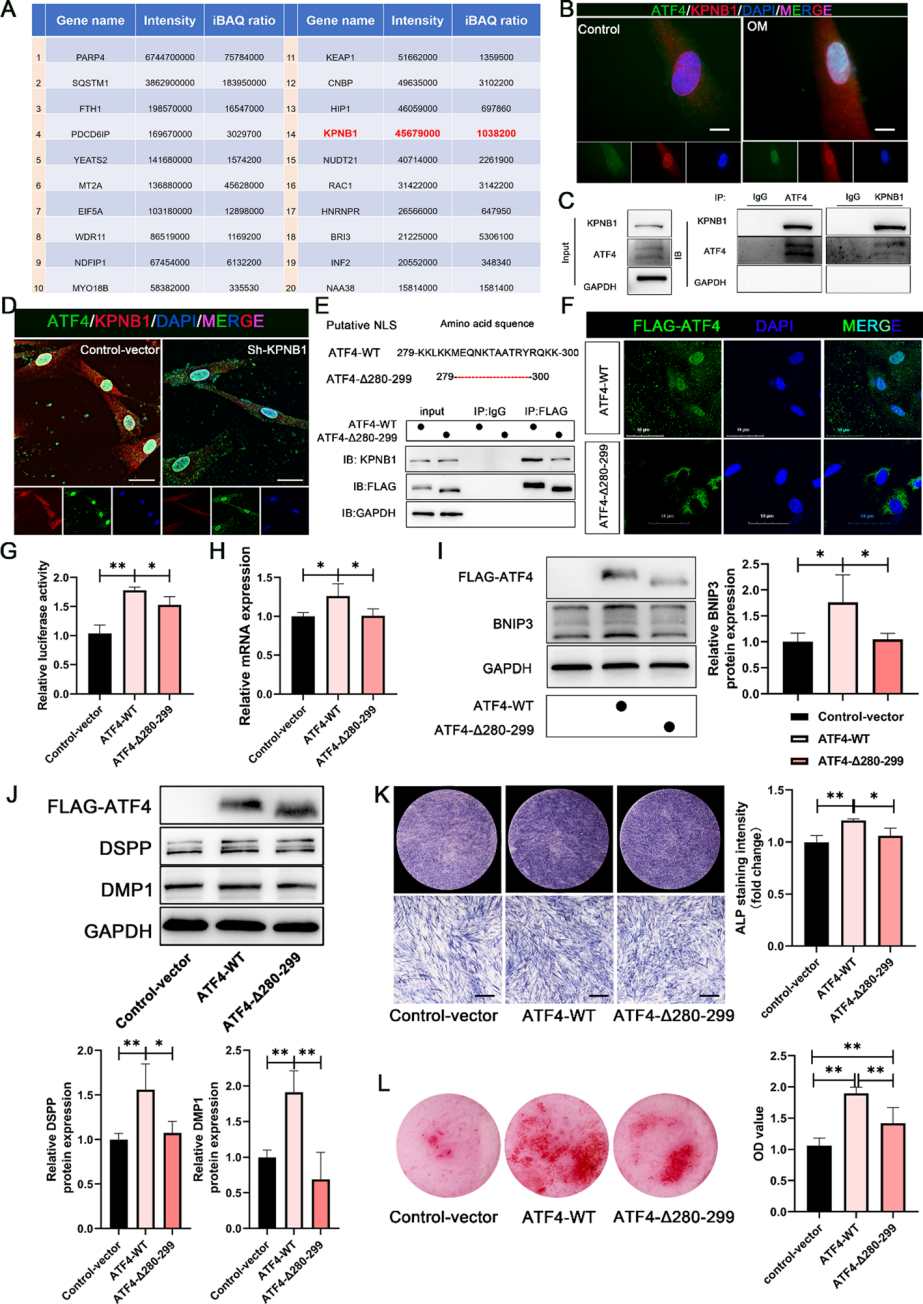
KPNB1 recognition of the NLS is essential for ATF4 translocation into the nucleus and DPSC differentiation into odontoblasts. **A** Mass spectrometry assays of potential ATF4-interacting proteins. **B** The cellular locations of ATF4 and KPNB1 were examined via confocal microscopy (scale bar: 20 μm). **C** Endogenous KPNB1 and ATF4 interactions were detected in DPSCs via the use of anti-KPNB1 and anti-ATF4 antibodies for Co-IP assays. **D** IF staining of ATF4 and KPNB1 localization after KPNB1 knockdown in DPSCs differentiated for 3 days (scale bar: 25 μm). **E** Putative NLS sequence segments of the ATF4 protein. The impact of the NLS mutation on the interaction between ATF4 and KPNB1 was detected via Co-IP assays. **F** IF images showing the localization of exogenously expressed wild-type ATF4 and a putative NLS mutant fused with FLAG in the DPSC differentiation process. Scale bar, 50 μm. **G** Dual-luciferase reporter experiments revealed showed that the putative NLS mutant plasmid was detrimental to *BNIP3* promoter transcription activity compared with the WT-ATF vector. **H** RT‒qPCR analysis revealed that compared with the WT-ATF4 vector, the putative NLS mutant plasmid weakened BNIP3 mRNA expression levels in DPSCs. **I** Western blot analysis revealed that, compared with the WT-ATF4 vector, the NLS mutant plasmid decreased BNIP3 protein expression levels in DPSCs. The right panel shows the quantitative representation of the western blot band intensities. **J**–**L** KPNB1-mediated nuclear import of ATF4 is needed for the osteogenic potential of DPSCs. **J** Odontoblastic marker expression levels were determined via western blot assays (7 days) after transfection with control/WT/NLS mutants fused with the FLAG-ATF4 vector. The lower panel shows the quantitative representation of the western blot band intensities. **K** ALP staining (7 days) of control/WT/NLS mutants fused with the FLAG-ATF4 vector. Scale bar, 500 μm. The right panel shows the semiquantification of ALP staining intensity. **L**, ARS staining (21 days) of control/WT/MUT ATF4 fused with the FLAG vector. The right panel shows the quantification of stained calcium deposits at 562 nm in DPSCs. The data are presented as the means ± SD. *N* ≥ 3; *P < 0.05 and **P < 0.01 indicate significant differences between the indicated columns

To elucidate the underlying mechanism of the nuclear import of ATF4, we performed mass spectrometry on DPSCs subjected to OM for 3 days and compared the results with those of control medium (Fig. S5). The primary goal of this analysis was to identify plausible interacting partners of ATF4 (Fig. 7A). Given that ATF4 accumulates mainly in the cell nucleus during DPSC differentiation, where it transcriptionally activates the target gene *BNIP3*, we observed a robust association between ATF4 and KPNB1, a molecular shuttle responsible for nucleocytoplasmic transport (Fig. 7B). This observation was further confirmed by a co-IP assay, which revealed an interaction between KPNB1 and ATF4, as both antibodies pulled down their respective target proteins (Fig. 7C). Moreover, KPNB1 knockdown (sh-KPNB1)-induced ATF4 protein signaling resulted in strong retention in the cytoplasm and weak expression in the cell nucleus during DPSC odontoblastic differentiation (Fig. S6A–C, Fig. 7D). However, there was no significant difference in KPNB1 expression between the DPSC differentiation group and the control group (Fig. S6D). In addition, knockdown of KPNB1 caused a significant decrease in *BNIP3* promoter transcriptional activity (Fig. S7A), BNIP3 expression (Fig. S7B-C), and DPSC odontoblastic differentiation (Fig. S7D-E). Consequently, KPNB1 recognition of ATF4 is crucial for promoting DPSC differentiation into odontoblast-like cells via ATF4.

KPNB1 is widely acknowledged to be responsible for shuttling cargo proteins between the cytoplasm and the cell nucleus, which relies on the NLS. Consequently, we analyzed the amino acid sequence of ATF4 and identified potential NLSs (Fig. 7E). We employed the sequence (280–299 amino acids [aa]) to construct a deletion mutation plasmid fused with FLAG for subsequent experiments. As shown in Fig. 7E, the co-IP assay revealed a significant decrease in the coprecipitation of KPNB1 with the ATF4-NLS mutant protein compared with the wild-type NLS protein. Additionally, IF staining with a FLAG tag revealed that the protein expressed by the full-length, wild-type ATF4 plasmid was predominantly localized within the nuclei of DPSCs. Conversely, upon deletion of the NLS sequence, ATF4 was sequestered in the cytoplasm and unable to translocate to the nucleus (Fig. 7F). Furthermore, compared with the wild-type ATF4 plasmid, the ATF4 NLS mutant failed to increase *BNIP3* transcriptional activity (Fig. 7G) or expression (Fig. 7H,I). These results collectively indicate that the NLS sequence (residues 280–299 aa) of ATF4 is crucial for KPNB1-mediated nuclear transport and transcriptional regulation of BNIP3.

**Fig. 8:**
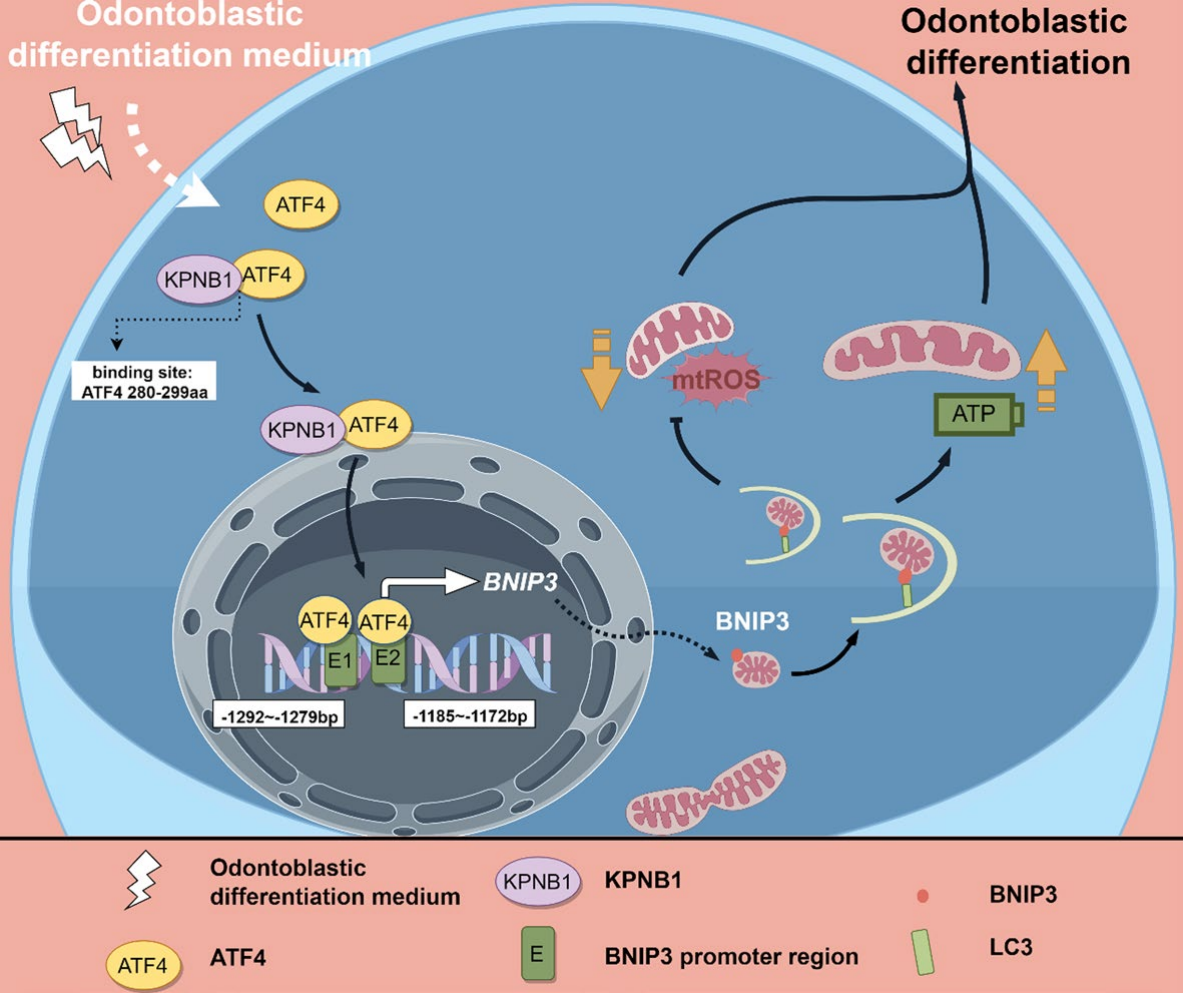
The diagram shows that KPNB1 recognizes the ATF4 nuclear localization sequence to assist in its transport into the nucleus, thereby transcriptionally activating the expression of BNIP3. This leads to increased mitophagy levels and optimized mitochondrial function, ultimately assisting DPSCs in odontogenic differentiation

To further investigate whether the NLS sequence of ATF4 contributes to the enhancement of DPSC differentiation, additional functional assays targeting the role of the NLS were performed. These findings revealed that the deletion of the NLS sequence from ATF4 caused the loss of its capacity to promote odontogenesis. This was evident in the results of western blotting, which was used to assess the expression of odontoblastic marker proteins (Fig. 7J), ALP staining (Fig. 7K), and ARS mineralized nodule staining (Fig. 7L) in DPSCs. In conclusion, these findings suggest that residues 280–299 of ATF4 directly interact with KPNB1, thereby modulating ATF4 nuclear import and its consequential effects on promoting *BNIP3* transcription and DPSC odontoblastic differentiation (Fig. 8).

## Discussion

With the advancements in tissue engineering, the regeneration of tooth tissue through the use of DPSCs has emerged as the most promising and exemplary reparative approach [33]. Nevertheless, the harsh microenvironment caused by inflammation, oxidative stress or nutritional deficiencies in the lesion area can greatly impair the activity and differentiation ability of graft stem cells [34–37]. Consequently, gaining profound insights into the intricate molecular regulatory mechanisms that steer DPSCs towards tooth differentiation is important for augmenting the clinical efficacy of stem cell applications [38]. Autophagy is an evolutionarily conserved degradation pathway in the cell that removes dysfunctional cytoplasmic components from lysosomes. If the process of autophagy is specific to the removal of damaged mitochondria, it is referred to as mitophagy [39]. The induction of mitophagy is important for promoting the differentiation of mesenchymal-derived stem cells towards osteogenesis and odontogenesis [40–43]. DPSCs exhibit a preference for glycolysis during proliferation but shift to oxidative phosphorylation when undergoing osteogenic differentiation [44, 45]. This process requires dramatic remodeling of the mitochondrial network, which involves both mitochondrial biogenesis and clearance. Hence, enhanced autophagy and mitophagy can not only create a favorable environment enriched with calcium and phosphorus vesicles but also supply high-quality mitochondria for cellular energy metabolism conducive to the differentiation process. In this study, we investigated whether BNIP3-dependent mitophagy is involved in the process of DPSC tooth differentiation and elucidated the pivotal molecules governing these transformative events. Additionally, we delved into the modulation of mitochondrial metabolism by these molecules and present novel perspectives on the avenues of development and therapeutic interventions.

Consistent with previous reports, this study demonstrated a significant enrichment of autophagy signaling pathways during DPSC tooth differentiation, as revealed through bioinformatics analysis and meticulous examination of autophagic flux [46]. Moreover, we noted increased mitophagy-related gene expression and increased colocalization of mitochondria with the autophagy marker protein LC3B, suggesting that mitophagy is significantly activated during the odontoblastic differentiation of DPSCs. Mitochondria are crucial organelles that control the regulation of glycolysis and oxidative phosphorylation fluxes for cell differentiation [47]. In the process of DPSC odontoblast cell differentiation, due to the increased energy demand of the cells and the increase in oxidative phosphorylation, the side effects lead to the production of excessive reactive oxygen species, which aggravate the generation of damaged mitochondria [44]. Therefore, mitophagy is necessary for continuous differentiation. Moreover, mitochondria are the main source and target of reactive oxygen species. Excessive accumulation of ROS can decrease mitochondrial membrane potential and mitochondrial dysfunction, leading to apoptosis. An increasing number of studies have shown that mitophagy is essential for the osteogenic/odontogenic differentiation of MSCS [48]. Bone marrow mesenchymal stem cells with mitochondrial damage exhibit low ALP activity and impaired formation of mineralized nodules, which is consistent with our findings that insufficient mitophagy hinders the odontogenic differentiation of DPSCs [49].

Mitophagy is usually mediated by ubiquitin-dependent pathways, PINK1/Parkin-dependent or receptor-mediated pathways, which include BNIP3, NIX, PHB1, and FUNDC1 [50]. All of these receptors contain long intergenic region sequences that bind LC3/GABARAP to degrade mitochondria via fusion with lysosomes [51]. BNIP3 has attracted our attention because of its significant upregulation of mitophagy-related DEGs. BNIP3 is a member of the apoptotic Bcl-2 protein family. BNIP3 plays a pivotal role in stem cell differentiation, particularly in cell populations abundant in mitochondria, such as cardiac progenitor cells, liver cells, and myoblasts [23, 52, 53]. Dysfunction of BNIP3-dependent mitophagy leads to increased intracellular mitochondrial superoxide levels and metabolic abnormalities [54–56]. In the present study, timeline-based protein expression analysis and Co-IP revealed high expression of BNIP3 during the odontoblastic differentiation of DPSCs, as well as direct binding to the lysosomal LC3B protein. BNIP3 knockdown impaired odontoblastic differentiation of DPSCs, and vice versa. The above results revealed for the first time that BNIP3-mediated mitophagy positively regulates the odontoblastic differentiation of DPSCs. Notably, mitophagy is a complex regulatory mechanism involving multiple protein- and organelle-related pathways. Under physiological conditions, mitophagy needs to be maintained at a certain level to participate in mitochondrial quality control. Abnormal mitophagy, such as excessive mitophagy, affects the health of the mitochondrial population, may lead to abnormal mitochondrial circulation and energy metabolism disorders, destroy the normal physiological function of cells, is harmful to cell survival, and can cause cell death [57]. Therefore, in tissue engineering applications, it is important to focus on the potential safety of mitophagy-based therapy, monitor molecules to confirm the homeostasis level of mitophagy, and determine how to achieve both seed cell function and survival.

Little attention has been given to the upstream regulatory pathways of mitophagy. Classic upstream regulatory factors of BNIP3 include FOXO3, HIF-1α, and BMAL1-CLOCK, which can directly bind to the *BNIP3* promoter, increasing the mitophagy in mesenchymal stem cells or embryonic stem cells [58–60]. In this study, we focused on a master stress-induced transcription factor named ATF4. On the one hand, our recent research and other studies [28, 61, 62] indicate that ATF4 acts as a pivotal promoter that drives the odontoblastic differentiation and function of DPSCs. On the one hand, ATF4 silencing blocks mitophagy induction both in vivo and in vitro [45]. Therefore, whether *BNIP3* can become the key target of ATF4 in influencing mitophagy has attracted our interest. Although research has shown that melatonin protects against environmental stress-induced fetal growth restriction, ATF4 and BNIP3 engage in mitophagy in placental trophoblasts through a positive relationship [29]. However, the specific upstream and downstream regulatory mechanisms between ATF4 and BNIP3 are ambiguous. In the present study, first, we verified the role of ATF4 in sustaining autophagic function and preventing mt-ROS in odontoblastic differentiation. Second, we showed that ATF4 directly binds to the approximately −1292 to  −1279 bp and approximately −1185 to −1172 bp regions of the *BNIP3* promoter to activate *BNIP3* transcription. This established the ATF4-BNIP3 signalling axis, providing a clearer understanding of the relationship between these two factors.

In addition, we further clarified the impact of mitophagy regulated by the ATF4-BNIP3 axis on mitochondrial function. These results confirmed that BNIP3 knockdown impaired the establishment of healthy mitochondrial function and differentiation outcomes mediated by ATF4. These findings indicate that the promotion of DPSC odontoblastic differentiation and mitochondrial stress resistance by ATF4 relies on BNIP3-dependent mitophagy via direct enhancement of *BNIP3* transcription. Interestingly, when ATF4 overexpression was accompanied by BNIP3 knockdown, both the level of cell differentiation and the maximum respiratory capacity of mitochondria were still greater than those in the double NC group, suggesting that ATF4 could act on not only BNIP3, but also other molecules related to mitophagy. Therefore, ATF4 is a key transcription factor that influences the regulation of mitochondrial activity during the differentiation of DPSCs, and its targets still need further investigation.

Transcription factors exert their transcriptional functions by being transported from the cytoplasm to the cell nucleus [63, 64]. Previous studies have shown that the activation of Toll-like receptor (TLR) signaling or ROS and IRE1α–PERK–eIF2α signaling in response to exogenous stimuli contributes to the entry of ATF4 into the nucleus [65, 66]. However, the molecular basis of ATF4 nuclear translocation remains ambiguous. To investigate the accompanying molecules involved in this process, we conducted IP‒MS experiments. These results suggest that KPNB1 strongly binds to ATF4 on the third day of DPSC odontoblastic differentiation. KPNB1 is a member of the karyopherin β family and mediates the transportation of proteins from the cytoplasm to the nucleus. Cyclin A, cyclin B1, cyclin D1, CDK4, and PD-L1 are known cargoes bound by KPNB1 [67, 68]. We hypothesized that ATF4 directly binds to the KPNB1 protein, facilitating the rapid nuclear translocation of ATF4. Subsequent IP experiments confirmed the direct binding between ATF4 and KPNB1, with binding occurring at amino acid residues 280–299 on ATF4. However, no upregulation of KPNB1 protein expression was detected in odontoblastic differentiated DPSCs. We speculate that the interaction between KPNB1 and ATF4 may be influenced by specific amino acid sequence modifications in diverse environments. This sequence contains many lysine residues, which are frequently modified and can impact protein function. Thus, the differences in the binding levels of KPNB1 and ATF4 may result from differentiation induction-induced lysine modifications. When KPNB1 is knocked down, it not only disrupts the essential odontoblastic differentiation potential of DPSCs but also leads to the accumulation of ATF4 in the cytoplasm, similar to the effect reported in glioma cells when KPNB1 is knocked down, causing endoplasmic reticulum (ER) stress and the upregulation of ATF4, an ER stress response molecule [69]. Furthermore, KPNB1, a crucial nuclear protein receptor, not only plays a role in ATF4-induced odontoblastic and osteogenic processes but also influences osteoblast maturation via continuous interactions with OXTRS, ARRBS, the small GTPase Rab5, and transportin-1 (TNPO1), facilitating OXTRS entry into the cell nucleus and ultimately affecting OXTRS-induced gene expression, which supports our findings as well [70]. Our data suggest that during DPSC odontoblastic differentiation, ATF4 nuclear translocation to promote *BNIP3* transcription is achieved through direct recognition of the NLS sequence (amino acids 280–299) of ATF4 by KPNB1. This process is essential for DPSC differentiation into odontoblasts. In addition, KPNB1 expression varies with the stage of the DPSC differentiation process or with the degree of differentiation of the cell population. The lack of cell population analysis in this study may explain why we were unable to detect differences in the expression of KPNB1 in the early stage of differentiation. Single-cell sequencing technology could be used to identify whether KPNB1 plays a specific role in the cell subclass according to the differentiation process of DPSCs in our future studies.

## Conclusion

In conclusion, considering the changes in mitochondrial function and broad metabolic alterations, our findings demonstrate that ATF4 binds to the promoter region (−1288 to 1217 bp and −1122 to 1087 bp) of *BNIP3* transcription and enhances BNIP3 expression in DPSCs undergoing odontoblastic differentiation. This process involves the interaction of ATF4 with KPNB1 through aa 280–299 for nuclear transport, indicating that the recognition of ATF4 by KPNB1 and the engagement of ATF4 in *BNIP3* transcription are crucial for regulating the mitochondrial function and odontoblast differentiation of DPSCs. Our study, which targets KPNB1/ATF4/BNIP3 axis-dependent mitophagy, may thus offer a promising therapeutic approach for stem cell therapy based on DPSCs.

## Supplementary Information

Supplementary materials 1: List of specific sequences of primers used in this study.

Supplementary materials 2: List of specific sequences of primers used for ChIP‒qPCR.

Supplementary materials 3: Figure 1. Isolation and characterization of DPSCs. A. Flow cytometric analysis revealed that DPSCs strongly positively expressed mesenchymal-associated markers, namely CD29 (99.8%), CD105 (99.6%), and CD146 (79.8%), whereas minimal expression of the hematopoietic markers CD14 (0.77%) and CD45 (0.57%) was detected. B. Alizarin red S staining of DPSCs induced odontoblastic differentiation after 21 days. Scale bar, 500 μm. C. Oil Red O staining after the adipogenic differentiation of DPSCs for 21 days. Scale bar, 50 μm. D. Alcian blue staining of DPSCs induced chondrogenic differentiation after 21 days. Scale bar, 200 μm. Figure 2. IF staining was used to assess the role of BNIP3 in DPSC differentiation into odontoblasts in vivo and the semiquantitative of BNIP3-, DMP1-, and DSPP-positive staining area intensities. Figure 3. Verification of the knockdown efficiency of BNIP3-siRNAs. Figure 4. IF staining was used to assess the role of ATF4/BNIP3 in DPSC differentiation into odontoblasts in vivo, and the semiquantitative intensities of the areas with positive staining for ATF4, BNIP3, DMP1, and DSPP were measured. Figure 5. Proteins that interact with ATF4 in DPSCs differentiate into odontoblasts. A. The gel was stained with Coomassie blue to visualize the total proteins bound to ATF4 in the control and OM medium. B. IP–MS results identifying ATF4-specific interacting proteins. Figure 6. Examination of the transfection efficiency of sh-KPNB1. A. Verification of the most efficient KPNB1 knockdown sequence in transfection experiments. B,C. Protein levels were determined by western blot analysis and normalized to that of GAPDH in terms of the relative intensity. D,E. KPNB1 expression in DPSCs treated with CON or OM for 3 days was determined by western blot analysis and normalized to that of GAPDH in terms of the relative intensity. Figure 7. KPNB1 knockdown impaired both BNIP3 expression and DPSC differentiation ability. A. dual luciferase reporter experiments were performed to assess BNIP3 promoter transcription. B. RT‒qPCR analysis of KPNB1 and BNIP3 mRNA expression levels in KPNB1-knockdown DPSCs. C. BNIP3 and odontoblastic marker expression levels were detected via western blotting (7 days) in DPSCs transfected with the control or sh-KPNB1 vector. The right panel shows the quantitative representation of the western blot band intensities. D. ALP staining (7 days) of the control/sh-KPNB1 vector-transfected cells. Scale bar, 500 μm. The right panel shows the semi-quantification of ALP staining intensity. E. ARS staining (21 days) with control/sh-KPNB1 vector. The right panel shows the quantification of stained calcium deposits at 562 nm in DPSCs. The data are presented as the means ± SD. *N* values from 3 to 8; *P < 0.05 and **P < 0.01 indicate significant differences between the indicated columns.

## Data Availability

The data presented in this study is available from the corresponding author on reasonable request.

## Abbreviations

ATF4: Activating transcription factor 4
ALP: Alkaline phosphatase
ARS: Alizarin red S
Baf A1: Bafilomycin A1
BNIP3: BCL2 protein-interacting protein 3
ChIP: Chromatin immunoprecipitation
DEG: Differentially expressed gene
DPSC: Dental pulp stem cell
EDTA: Ethylenediaminetetraacetic acid
FCCP: Carbonyl cyanide p-trifluoromethoxyphenylhydrazone
HE: Hematoxylin–eosin
IF: Immunofluorescence
IHC: Immunohistochemistry
KPNB1: Importin subunit beta-1
LC3BI: Microtubule-associated protein 1 light chain 3 beta I
LC3BII: Microtubule-associated protein 1 light chain 3 beta II
MOI: Multiplicity of infection
mtROS: Mitochondrial reactive oxygen species
NLS: Nuclear localization signal
OCR: Oxygen consumption rate
Oligo: Oligomycin
P62: Ubiquitin-binding protein p62; sequestosome-1
R/A: Rotenone/antimycinA
Rapa: Rapamycin

## References

[1] Sui B, Wu D, Xiang L, Fu Y, Kou X, Shi S. Dental pulp stem cells: from discovery to clinical application. J Endod. 2020;46(9S):S46–55. 32950195 10.1016/j.joen.2020.06.027

[2] Stefanska K, Volponi AA, Kulus M, Wasko J, Farzaneh M, Grzelak J, et al. Dental pulp stem cells - a basic research and future application in regenerative medicine. Biomed Pharmacother. 2024;178: 116990. 39024839 10.1016/j.biopha.2024.116990

[3] Lei M, Li K, Li B, Gao LN, Chen FM, Jin Y. Mesenchymal stem cell characteristics of dental pulp and periodontal ligament stem cells after in vivo transplantation. Biomaterials. 2014;35(24):6332–43. 24824581 10.1016/j.biomaterials.2014.04.071

[4] Naz S, Khan FR, Khan I, Zohra RR, Salim A, Mohammed N, et al. Comparative analysis of dental pulp stem cells and stem cells from human exfoliated teeth in terms of growth kinetics, immunophenotype, self-renewal and multi lineage differentiation potential for future perspective of calcified tissue regeneration. Pak J Med Sci. 2022;38(5):1228–37. 35799722 10.12669/pjms.38.5.5187 PMC9247794

[5] Lopez-Garcia S, Aznar-Cervantes SD, Pagan A, Llena C, Forner L, Sanz JL, et al. 3D Graphene/silk fibroin scaffolds enhance dental pulp stem cell osteo/odontogenic differentiation. Dent Mater. 2024;40(3):431–40. 38114344 10.1016/j.dental.2023.12.009

[6] Caputi S, Trubiani O, Sinjari B, Trofimova S, Diomede F, Linkova N, et al. Effect of short peptides on neuronal differentiation of stem cells. Int J Immunopathol Pharmacol. 2019;33:2058738419828613. 30791821 10.1177/2058738419828613 PMC6376556

[7] Diomede F, Marconi GD, Guarnieri S, D’Attilio M, Cavalcanti M, Mariggio MA, et al. A novel role of ascorbic acid in anti-inflammatory pathway and ROS generation in HEMA treated dental pulp stem cells. Materials (Basel). 2019. 10.3390/ma13010130. 31892218 10.3390/ma13010130 PMC6981406

[8] Krivanek J, Soldatov RA, Kastriti ME, Chontorotzea T, Herdina AN, Petersen J, et al. Dental cell type atlas reveals stem and differentiated cell types in mouse and human teeth. Nat Commun. 2020;11(1):4816. 32968047 10.1038/s41467-020-18512-7 PMC7511944

[9] Itoh Y, Sasaki JI, Hashimoto M, Katata C, Hayashi M, Imazato S. Pulp regeneration by 3-dimensional dental pulp stem cell constructs. J Dent Res. 2018;97(10):1137–43. 29702010 10.1177/0022034518772260

[10] Sanz JL, Soler-Doria A, Lopez-Garcia S, Garcia-Bernal D, Rodriguez-Lozano FJ, Lozano A, et al. Comparative biological properties and mineralization potential of 3 endodontic materials for vital pulp therapy: theracal PT, theracal LC, and biodentine on human dental pulp stem cells. J Endod. 2021;47(12):1896–906. 34425148 10.1016/j.joen.2021.08.001

[11] Liang C, Liao L, Tian W. Stem cell-based dental pulp regeneration: insights from signaling pathways. Stem Cell Rev Rep. 2021;17(4):1251–63. 33459973 10.1007/s12015-020-10117-3

[12] Li Z, Wu M, Liu S, Liu X, Huan Y, Ye Q, et al. Apoptotic vesicles activate autophagy in recipient cells to induce angiogenesis and dental pulp regeneration. Mol Ther. 2022;30(10):3193–208. 35538661 10.1016/j.ymthe.2022.05.006 PMC9552912

[13] Lu J, Li R, Ni S, Xie Y, Liu X, Zhang K, et al. Metformin carbon nanodots promote odontoblastic differentiation of dental pulp stem cells by pathway of autophagy. Front Bioeng Biotechnol. 2022;10:1002291. 36159662 10.3389/fbioe.2022.1002291 PMC9506707

[14] Pei DD, Sun JL, Zhu CH, Tian FC, Jiao K, Anderson MR, et al. Contribution of mitophagy to cell-mediated mineralization: revisiting a 50-year-old conundrum. Adv Sci (Weinh). 2018;5(10):1800873. 30356983 10.1002/advs.201800873 PMC6193168

[15] Du H, Wolf J, Schafer B, Moldoveanu T, Chipuk JE, Kuwana T. BH3 domains other than Bim and Bid can directly activate Bax/Bak. J Biol Chem. 2011;286(1):491–501. 21041309 10.1074/jbc.M110.167148 PMC3013008

[16] Yan W, Diao S, Fan Z. The role and mechanism of mitochondrial functions and energy metabolism in the function regulation of the mesenchymal stem cells. Stem Cell Res Ther. 2021;12(1):140. 33597020 10.1186/s13287-021-02194-z PMC7890860

[17] Teresak P, Lapao A, Subic N, Boya P, Elazar Z, Simonsen A. Regulation of PRKN-independent mitophagy. Autophagy. 2022;18(1):24–39. 33570005 10.1080/15548627.2021.1888244 PMC8865282

[18] Lee SY, An HJ, Kim JM, Sung MJ, Kim DK, Kim HK, et al. PINK1 deficiency impairs osteoblast differentiation through aberrant mitochondrial homeostasis. Stem Cell Res Ther. 2021;12(1):589. 34823575 10.1186/s13287-021-02656-4 PMC8614054

[19] Cai C, Guo Z, Chang X, Li Z, Wu F, He J, et al. Empagliflozin attenuates cardiac microvascular ischemia/reperfusion through activating the AMPKalpha1/ULK1/FUNDC1/mitophagy pathway. Redox Biol. 2022;52: 102288. 35325804 10.1016/j.redox.2022.102288 PMC8938627

[20] Choi GE, Lee HJ, Chae CW, Cho JH, Jung YH, Kim JS, et al. BNIP3L/NIX-mediated mitophagy protects against glucocorticoid-induced synapse defects. Nat Commun. 2021;12(1):487. 33473105 10.1038/s41467-020-20679-y PMC7817668

[21] Lin L, Li S, Hu S, Yu W, Jiang B, Mao C, et al. UCHL1 impairs periodontal ligament stem cell osteogenesis in periodontitis. J Dent Res. 2023;102(1):61–71. 36112902 10.1177/00220345221116031

[22] Chen Y, Jiao D, Liu Y, Xu X, Wang Y, Luo X, et al. FBXL4 mutations cause excessive mitophagy via BNIP3/BNIP3L accumulation leading to mitochondrial DNA depletion syndrome. Cell Death Differ. 2023;30(10):2351–63. 37568009 10.1038/s41418-023-01205-1 PMC10589232

[23] Baechler BL, Bloemberg D, Quadrilatero J. Mitophagy regulates mitochondrial network signaling, oxidative stress, and apoptosis during myoblast differentiation. Autophagy. 2019;15(9):1606–19. 30859901 10.1080/15548627.2019.1591672 PMC6693454

[24] Zhao Q, Liu K, Zhang L, Li Z, Wang L, Cao J, et al. BNIP3-dependent mitophagy safeguards ESC genomic integrity via preventing oxidative stress-induced DNA damage and protecting homologous recombination. Cell Death Dis. 2022;13(11):976. 36402748 10.1038/s41419-022-05413-4 PMC9675825

[25] Zhang Y, Lin T, Lian N, Tao H, Li C, Li L, et al. Hop2 interacts with ATF4 to promote osteoblast differentiation. J Bone Miner Res. 2019;34(12):2287–300. 31433867 10.1002/jbmr.3857 PMC7422940

[26] Xiao Y, Xie X, Chen Z, Yin G, Kong W, Zhou J. Advances in the roles of ATF4 in osteoporosis. Biomed Pharmacother. 2023;169: 115864. 37948991 10.1016/j.biopha.2023.115864

[27] Wang X, Guo B, Li Q, Peng J, Yang Z, Wang A, et al. miR-214 targets ATF4 to inhibit bone formation. Nat Med. 2013;19(1):93–100. 23223004 10.1038/nm.3026

[28] Yu S, Guo J, Yang D, Yan X, Zhang Z, Wei P, et al. The ATF4-regulated LncRNA MALAT1 promotes odontoblastic differentiation of human dental pulp stem cells via histone demethylase JMJD3: an in vitro study. Int Endod J. 2024;57(1):50–63. 37837219 10.1111/iej.13984

[29] Zhu HL, Shi XT, Xu XF, Zhou GX, Xiong YW, Yi SJ, et al. Melatonin protects against environmental stress-induced fetal growth restriction via suppressing ROS-mediated GCN2/ATF4/BNIP3-dependent mitophagy in placental trophoblasts. Redox Biol. 2021;40: 101854. 33454563 10.1016/j.redox.2021.101854 PMC7811044

[30] Pan XC, Xiong YL, Hong JH, Liu Y, Cen YY, Liu T, et al. Cardiomyocytic FoxP3 is involved in Parkin-mediated mitophagy during cardiac remodeling and the regulatory role of triptolide. Theranostics. 2022;12(5):2483–501. 35265221 10.7150/thno.71102 PMC8899572

[31] Zhang B, Xiao M, Cheng X, Bai Y, Chen H, Yu Q, et al. Enamel matrix derivative enhances the odontoblastic differentiation of dental pulp stem cells via activating MAPK signaling pathways. Stem Cells Int. 2022;2022:2236250. 35530415 10.1155/2022/2236250 PMC9071913

[32] Zhang Z, Bao Y, Wei P, Yan X, Qiu Q, Qiu L. Melatonin attenuates dental pulp stem cells senescence due to vitro expansion via inhibiting MMP3. Oral Dis. 2024;30(4):2410–24. 37448325 10.1111/odi.14649

[33] Sui BD, Zheng CX, Zhao WM, Xuan K, Li B, Jin Y. Mesenchymal condensation in tooth development and regeneration: a focus on translational aspects of organogenesis. Physiol Rev. 2023;103(3):1899–964. 36656056 10.1152/physrev.00019.2022

[34] Cui Y, Ji W, Gao Y, Xiao Y, Liu H, Chen Z. Single-cell characterization of monolayer cultured human dental pulp stem cells with enhanced differentiation capacity. Int J Oral Sci. 2021;13(1):44. 34911932 10.1038/s41368-021-00140-6 PMC8674359

[35] Wang Y, Ma D, Wu Z, Yang B, Li R, Zhao X, et al. Clinical application of mesenchymal stem cells in rheumatic diseases. Stem Cell Res Ther. 2021;12(1):567. 34753496 10.1186/s13287-021-02635-9 PMC8579678

[36] Sriram S, Yuan C, Chakraborty S, Tay W, Park M, Shabbir A, et al. Oxidative stress mediates depot-specific functional differences of human adipose-derived stem cells. Stem Cell Res Ther. 2019;10(1):141. 31113471 10.1186/s13287-019-1240-y PMC6528291

[37] Cui S, Liu X, Liu Y, Hu W, Ma K, Huang Q, et al. Autophagosomes defeat ferroptosis by decreasing generation and increasing discharge of free Fe(2+) in skin repair cells to accelerate diabetic wound healing. Adv Sci (Weinh). 2023;10(25): e2300414. 37387572 10.1002/advs.202300414 PMC10477857

[38] Guo Y, Chi X, Wang Y, Heng BC, Wei Y, Zhang X, et al. Mitochondria transfer enhances proliferation, migration, and osteogenic differentiation of bone marrow mesenchymal stem cell and promotes bone defect healing. Stem Cell Res Ther. 2020;11(1):245. 32586355 10.1186/s13287-020-01704-9 PMC7318752

[39] Gustafsson AB, Dorn GW 2nd. Evolving and expanding the roles of Mitophagy as a homeostatic and pathogenic process. Physiol Rev. 2019;99(1):853–92. 30540226 10.1152/physrev.00005.2018 PMC6442924

[40] Yang JW, Zhang YF, Wan CY, Sun ZY, Nie S, Jian SJ, et al. Autophagy in SDF-1alpha-mediated DPSC migration and pulp regeneration. Biomaterials. 2015;44:11–23. 25617122 10.1016/j.biomaterials.2014.12.006

[41] Zhan Y, Wang H, Zhang L, Pei F, Chen Z. HDAC6 regulates the fusion of autophagosome and lysosome to involve in odontoblast differentiation. Front Cell Dev Biol. 2020;8: 605609. 33330506 10.3389/fcell.2020.605609 PMC7732691

[42] Chen L, Shi X, Xie J, Weng SJ, Xie ZJ, Tang JH, et al. Apelin-13 induces mitophagy in bone marrow mesenchymal stem cells to suppress intracellular oxidative stress and ameliorate osteoporosis by activation of AMPK signaling pathway. Free Radic Biol Med. 2021;163:356–68. 33385540 10.1016/j.freeradbiomed.2020.12.235

[43] Iwayama T, Bhongsatiern P, Takedachi M, Murakami S. Matrix vesicle-mediated mineralization and potential applications. J Dent Res. 2022;101(13):1554–62. 35722955 10.1177/00220345221103145

[44] Guo J, Zhou F, Liu Z, Cao Y, Zhao W, Zhang Z, et al. Exosome-shuttled mitochondrial transcription factor A mRNA promotes the osteogenesis of dental pulp stem cells through mitochondrial oxidative phosphorylation activation. Cell Prolif. 2022;55(12): e13324. 36054692 10.1111/cpr.13324 PMC9715363

[45] Wan L, Wang L, Cheng R, Cheng L, Hu T. Metabolic shift and the effect of mitochondrial respiration on the osteogenic differentiation of dental pulp stem cells. PeerJ. 2023;11: e15164. 37101792 10.7717/peerj.15164 PMC10124543

[46] Yin Y, Tian BM, Li X, Yu YC, Deng DK, Sun LJ, et al. Gold nanoparticles targeting the autophagy-lysosome system to combat the inflammation-compromised osteogenic potential of periodontal ligament stem cells: from mechanism to therapy. Biomaterials. 2022;288: 121743. 36030103 10.1016/j.biomaterials.2022.121743

[47] Li X, Tian BM, Deng DK, Liu F, Zhou H, Kong DQ, et al. LncRNA GACAT2 binds with protein PKM1/2 to regulate cell mitochondrial function and cementogenesis in an inflammatory environment. Bone Res. 2022;10(1):29. 35296649 10.1038/s41413-022-00197-x PMC8927299

[48] Liu F, Yuan Y, Bai L, Yuan L, Li L, Liu J, et al. LRRc17 controls BMSC senescence via mitophagy and inhibits the therapeutic effect of BMSCs on ovariectomy-induced bone loss. Redox Biol. 2021;43: 101963. 33865167 10.1016/j.redox.2021.101963 PMC8066428

[49] Shen Y, Wu L, Qin D, Xia Y, Zhou Z, Zhang X, et al. Carbon black suppresses the osteogenesis of mesenchymal stem cells: the role of mitochondria. Part Fibre Toxicol. 2018;15(1):16. 29650039 10.1186/s12989-018-0253-5 PMC5897950

[50] Ni HM, Williams JA, Ding WX. Mitochondrial dynamics and mitochondrial quality control. Redox Biol. 2015;4:6–13. 25479550 10.1016/j.redox.2014.11.006 PMC4309858

[51] He YL, Li J, Gong SH, Cheng X, Zhao M, Cao Y, et al. BNIP3 phosphorylation by JNK1/2 promotes mitophagy via enhancing its stability under hypoxia. Cell Death Dis. 2022;13(11):966. 36396625 10.1038/s41419-022-05418-z PMC9672126

[52] Lampert MA, Orogo AM, Najor RH, Hammerling BC, Leon LJ, Wang BJ, et al. BNIP3L/NIX and FUNDC1-mediated mitophagy is required for mitochondrial network remodeling during cardiac progenitor cell differentiation. Autophagy. 2019;15(7):1182–98. 30741592 10.1080/15548627.2019.1580095 PMC6613840

[53] Glick D, Zhang W, Beaton M, Marsboom G, Gruber M, Simon MC, et al. BNip3 regulates mitochondrial function and lipid metabolism in the liver. Mol Cell Biol. 2012;32(13):2570–84. 22547685 10.1128/MCB.00167-12 PMC3434502

[54] Vara-Perez M, Rossi M, Van den Haute C, Maes H, Sassano ML, Venkataramani V, et al. BNIP3 promotes HIF-1alpha-driven melanoma growth by curbing intracellular iron homeostasis. EMBO J. 2021;40(10): e106214. 33932034 10.15252/embj.2020106214 PMC8126921

[55] Tang C, Han H, Liu Z, Liu Y, Yin L, Cai J, et al. Activation of BNIP3-mediated mitophagy protects against renal ischemia-reperfusion injury. Cell Death Dis. 2019;10(9):677. 31515472 10.1038/s41419-019-1899-0 PMC6742651

[56] Madhu V, Hernandez-Meadows M, Boneski PK, Qiu Y, Guntur AR, Kurland IJ, et al. The mitophagy receptor BNIP3 is critical for the regulation of metabolic homeostasis and mitochondrial function in the nucleus pulposus cells of the intervertebral disc. Autophagy. 2023;19(6):1821–43. 36628478 10.1080/15548627.2022.2162245 PMC10262801

[57] Picca A, Faitg J, Auwerx J, Ferrucci L, D’Amico D. Mitophagy in human health, ageing and disease. Nat Metab. 2023;5(12):2047–61. 38036770 10.1038/s42255-023-00930-8 PMC12159423

[58] Li E, Li X, Huang J, Xu C, Liang Q, Ren K, et al. BMAL1 regulates mitochondrial fission and mitophagy through mitochondrial protein BNIP3 and is critical in the development of dilated cardiomyopathy. Protein Cell. 2020;11(9):661–79. 32277346 10.1007/s13238-020-00713-x PMC7452999

[59] Lee HJ, Jung YH, Choi GE, Ko SH, Lee SJ, Lee SH, et al. BNIP3 induction by hypoxia stimulates FASN-dependent free fatty acid production enhancing therapeutic potential of umbilical cord blood-derived human mesenchymal stem cells. Redox Biol. 2017;13:426–43. 28704726 10.1016/j.redox.2017.07.004 PMC5508529

[60] Kothari S, Cizeau J, McMillan-Ward E, Israels SJ, Bailes M, Ens K, et al. BNIP3 plays a role in hypoxic cell death in human epithelial cells that is inhibited by growth factors EGF and IGF. Oncogene. 2003;22(30):4734–44. 12879018 10.1038/sj.onc.1206666

[61] Jia L, Li D, Wang YN, Zhang D, Xu X. PSAT1 positively regulates the osteogenic lineage differentiation of periodontal ligament stem cells through the ATF4/PSAT1/Akt/GSK3beta/beta-catenin axis. J Transl Med. 2023;21(1):70. 36732787 10.1186/s12967-022-03775-z PMC9893676

[62] Yang S, Hu L, Wang C, Wei F. PERK-eIF2alpha-ATF4 signaling contributes to osteogenic differentiation of periodontal ligament stem cells. J Mol Histol. 2020;51(2):125–35. 32124153 10.1007/s10735-020-09863-y

[63] Park JW, Lee EJ, Moon E, Kim HL, Kim IB, Hodzic D, et al. Orthodenticle homeobox 2 is transported to lysosomes by nuclear budding vesicles. Nat Commun. 2023;14(1):1111. 36849521 10.1038/s41467-023-36697-5 PMC9971051

[64] Choo HJ, Cutler A, Rother F, Bader M, Pavlath GK. Karyopherin alpha 1 regulates satellite cell proliferation and survival by modulating nuclear import. Stem Cells. 2016;34(11):2784–97. 27434733 10.1002/stem.2467 PMC5247404

[65] Lan JF, Zhao LJ, Wei S, Wang Y, Lin L, Li XC. PcToll2 positively regulates the expression of antimicrobial peptides by promoting PcATF4 translocation into the nucleus. Fish Shellfish Immunol. 2016;58:59–66. 27623341 10.1016/j.fsi.2016.09.007

[66] Yang B, Xu Y, Hu Y, Luo Y, Lu X, Tsui CK, et al. Madecassic Acid protects against hypoxia-induced oxidative stress in retinal microvascular endothelial cells via ROS-mediated endoplasmic reticulum stress. Biomed Pharmacother. 2016;84:845–52. 27728894 10.1016/j.biopha.2016.10.015

[67] Du W, Zhu J, Zeng Y, Liu T, Zhang Y, Cai T, et al. KPNB1-mediated nuclear translocation of PD-L1 promotes non-small cell lung cancer cell proliferation via the Gas6/MerTK signaling pathway. Cell Death Differ. 2021;28(4):1284–300. 33139930 10.1038/s41418-020-00651-5 PMC8027631

[68] Shi Q, Lin M, Cheng X, Zhang Z, Deng S, Lang K, et al. KPNB1-mediated nuclear import in cancer. Eur J Pharmacol. 2023;955: 175925. 37473981 10.1016/j.ejphar.2023.175925

[69] Zhu ZC, Liu JW, Li K, Zheng J, Xiong ZQ. KPNB1 inhibition disrupts proteostasis and triggers unfolded protein response-mediated apoptosis in glioblastoma cells. Oncogene. 2018;37(22):2936–52. 29520102 10.1038/s41388-018-0180-9 PMC5978811

[70] Di Benedetto A, Sun L, Zambonin CG, Tamma R, Nico B, Calvano CD, et al. Osteoblast regulation via ligand-activated nuclear trafficking of the oxytocin receptor. Proc Natl Acad Sci U S A. 2014;111(46):16502–7. 25378700 10.1073/pnas.1419349111 PMC4246276

