# Supplementary material for: KPNB1-ATF4 induces BNIP3-dependent mitophagy to drive odontoblastic differentiation in dental pulp stem cells
Source: Cell Mol Biol Lett. 2024 Nov 27;29:145. doi: 10.1186/s11658-024-00664-9 (PMC11600598; doi:10.1186/s11658-024-00664-9)
Supplement: Supplementary file 1 — Supplementary materials 1: List of specific sequences of primers used in this study. [file 11658_2024_664_MOESM1_ESM.docx]

**Supplementary file 1. List of specific sequences of primers used in this study.**

| **Gene name** | **Primer’s sequence (5’-3’)** |
| --- | --- |
| *GAPDH* | F: ACAGCCTGGATAGCAACG; R: GGTCATGAGTCCTTCCACGATACC |
| *β ACTIN* | F: CACCAACTGGGACGACAT; R: ACAGCCTGGATAGCAACG |
| *ATF4* | F: CCCTTCACCTTCTTACAACCTC; R: TGCCCAGCTCTAAACTAAAGGA |
| *BNIP3* | F: CTGGACGGAGTAGCTCCAAG; R: CCGACTTGACCAATCCCATA |
| *KPNB1* | F: TGTGATGCTGGTACAACCCA; R: TAGTCTTCGATCTCCGCCCT |
| *BNIP1* | F: CATGCAGTCTCTAGTCACTTCT; R: ATAGAGGACCGTAGCAAGAAAC |
| *IRGM* | F: TGGACCAAGCTAGACATGGACCTC; R: GTATTCACATACCCGCTCCTTCTGG |
| *BAG3* | F: AGAGACGGTGTCAGGAAGGTTCAG; R: GTTGCTGGGCTGGAGTTCATAGAC |
| *MAP1LC3B* | F: GTCAGCGTCTCCACACCAATCTC R: ACAATTTCATCCCGAACGTCTCCTG |
| *HDAC6* | F: GTGTCACTTCGAAGCGAAATAT; R: CCACGATTAGGTCTTCTTCCAT |
| *PRKAR1A* | F: CTCTACGTCCAGAAGCATAACA; R: CCAACCTCTCAAAGTATTCCCT |
| *ERN1* | F: CGTGAGCGACAGAATAGAAAAG; R: GCTTCTTATTTCTCATGGCTCG |
| *PPP1R15A* | F: GATGAGGATGTGGATAGTGAGG; R: CTGTCTCTTTTCCAGGTCGATA |
| *PIK3R4* | F: GCTCGTCAAATAAGTACAGCTG; R: AACCATTTCGTTTCTTCTGACG |
| *CTSB* | F: ATACTCAGAGGACAGGATCACT; R: ATCTTTTCCCAGTACTGATCGG |
| *SQSTM1* | F: TGATTGAGTCCCTCTCCCAGATGC; R: CCGCTCCGATGTCATAGTTCTTGG |
| *RHEB* | F: GCTTTGGCAGAATCTTGGAATG; R: GACTTGCCTTGTGAAGCTGCC |

|  |  |  |
| --- | --- | --- |
