# Supplementary material for: KPNB1-ATF4 induces BNIP3-dependent mitophagy to drive odontoblastic differentiation in dental pulp stem cells
Source: Cell Mol Biol Lett. 2024 Nov 27;29:145. doi: 10.1186/s11658-024-00664-9 (PMC11600598; doi:10.1186/s11658-024-00664-9)
Supplement: Supplementary file 2 — Supplementary materials 2: List of specific sequences of primers used for ChIP‒qPCR. [file 11658_2024_664_MOESM2_ESM.docx]

**Supplementary file 2. List of specific sequences of primers used for ChIP‒ qPCR.**

| **Gene name** | **Primer’s sequence (5’-3’)** |
| --- | --- |
| *Potential binding site 1*  *-1292~-1279* | F: GAATCCTCCCACAGCAGGT; R: GTGGGGCGCATGTCTTTC |
| *Potential binding site 2*  *-1185~-1172* | F: CACACTGGCCTCTGGATGT; R: AGAAGCTGAACCGGGGTC |
| *Potential binding site 3* |  |
| *+107~+120* | F: ACCTCCGCTTTCCCACC; R: CGCTCCGTTCTGCGACAT |

|  |  |  |
| --- | --- | --- |
