# Supplementary material for: KPNB1-ATF4 induces BNIP3-dependent mitophagy to drive odontoblastic differentiation in dental pulp stem cells
Source: Cell Mol Biol Lett. 2024 Nov 27;29:145. doi: 10.1186/s11658-024-00664-9 (PMC11600598; doi:10.1186/s11658-024-00664-9)
Supplement: Supplementary file 3 — Supplementary materials 3: Figure 1. Isolation and characterization of DPSCs. A. Flow cytometric analysis revealed that DPSCs strongly positively expressed mesenchymal-associated markers, namely CD29 (99.8%), CD105 (99.6%), and CD146 (79.8%), whereas minimal expression of the hematopoietic markers CD14 (0.77%) and CD45 (0.57%) was detected. B. Alizarin red S staining of DPSCs induced odontoblastic differentiation after 21 days. Scale bar, 500 μm. C. Oil Red O staining after the adipogenic differentiation of DPSCs for 21 days. Scale bar, 50 μm. D. Alcian blue staining of DPSCs induced chondrogenic differentiation after 21 days. Scale bar, 200 μm. Figure 2. IF staining was used to assess the role of BNIP3 in DPSC differentiation into odontoblasts in vivo and the semiquantitative of BNIP3-, DMP1-, and DSPP-positive staining area intensities. Figure 3. Verification of the knockdown efficiency of BNIP3-siRNAs. Figure 4. IF staining was used to assess the role of ATF4/BNIP3 in DPSC differentiation into odontoblasts in vivo, and the semiquantitative intensities of the areas with positive staining for ATF4, BNIP3, DMP1, and DSPP were measured. Figure 5. Proteins that interact with ATF4 in DPSCs differentiate into odontoblasts. A. The gel was stained with Coomassie blue to visualize the total proteins bound to ATF4 in the control and OM medium. B. IP–MS results identifying ATF4-specific interacting proteins. Figure 6. Examination of the transfection efficiency of sh-KPNB1. A. Verification of the most efficient KPNB1 knockdown sequence in transfection experiments. B,C. Protein levels were determined by western blot analysis and normalized to that of GAPDH in terms of the relative intensity. D,E. KPNB1 expression in DPSCs treated with CON or OM for 3 days was determined by western blot analysis and normalized to that of GAPDH in terms of the relative intensity. Figure 7. KPNB1 knockdown impaired both BNIP3 expression and DPSC differentiation ability. A. dual luciferase reporte [file 11658_2024_664_MOESM3_ESM.docx]

**Supplementary figures**

**Supplementary figure 1**


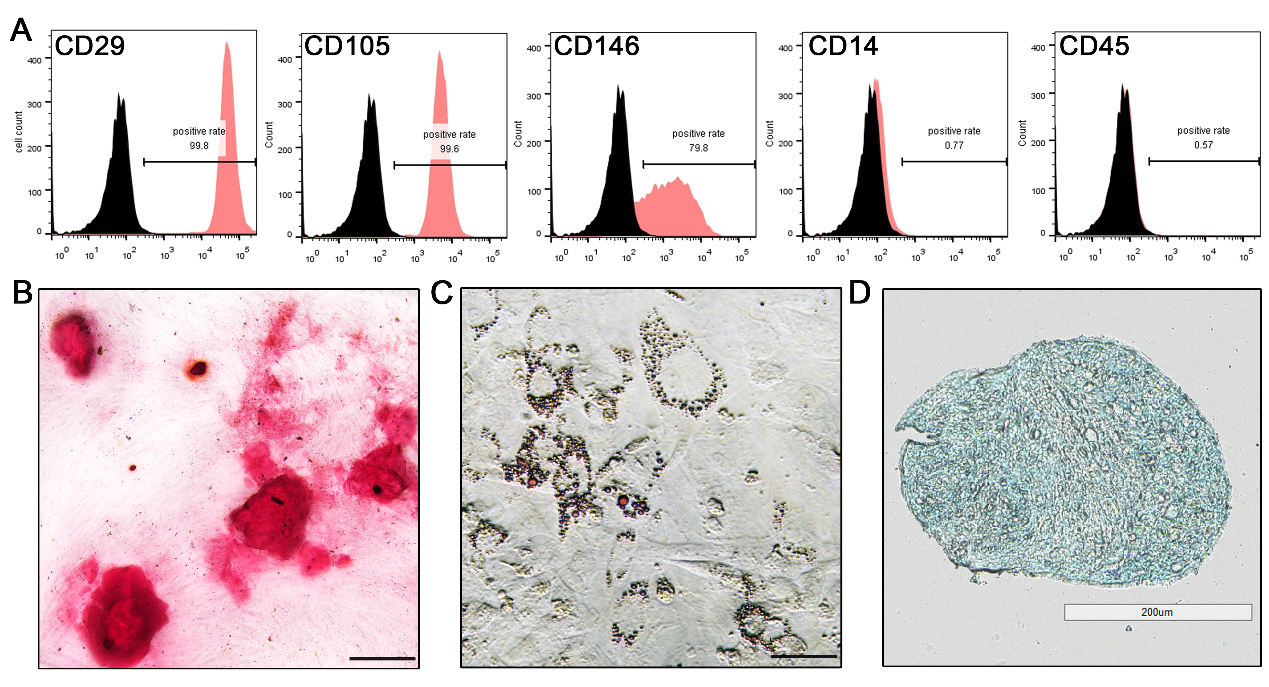
**Supplementary Figure 1. Isolation and characterization of DPSCs.**

A. Flow cytometric analysis revealed that DPSCs strongly positively expressed mesenchymal-associated markers, namely, CD29 (99.8%), CD105 (99.6%), and CD146 (79.8%), whereas minimal expression of the haematopoietic markers CD14 (0.77%) and CD45 (0.57%) was detected.

B. Alizarin red S staining of DPSCs induced odontoblastic differentiation after 21 days. Scale bar= 500 μm.

C. Oil Red O staining after the adipogenic differentiation of DPSCs for 21 days. Scale bar, 50 μm.

D. Alcian blue staining of DPSCs induced chondrogenic differentiation after 21 days. Scale bar, 200 μm.


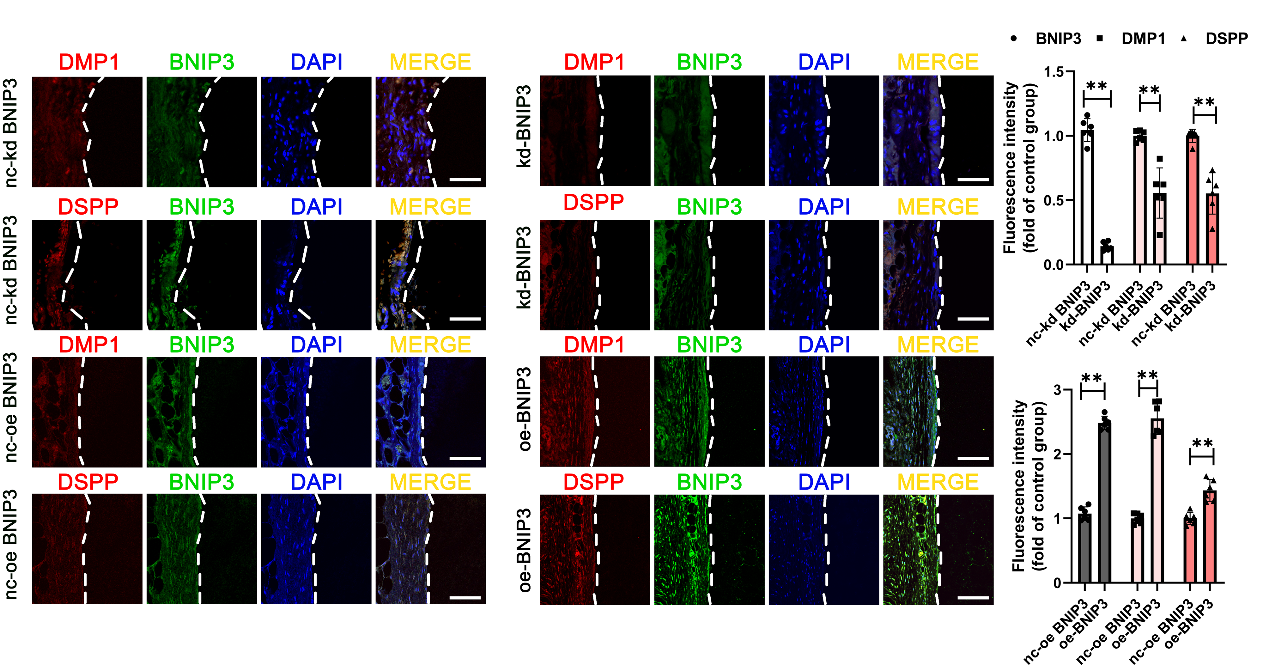
**Supplementary figure 2**

**Supplementary Figure 2. IF staining was used to assess the role of BNIP3 in DPSC differentiation into odontoblasts in vivo and the semiquantitative of BNIP3-, DMP1-, and DSPP-positive staining area intensities**.

**Supplementary figure 3**


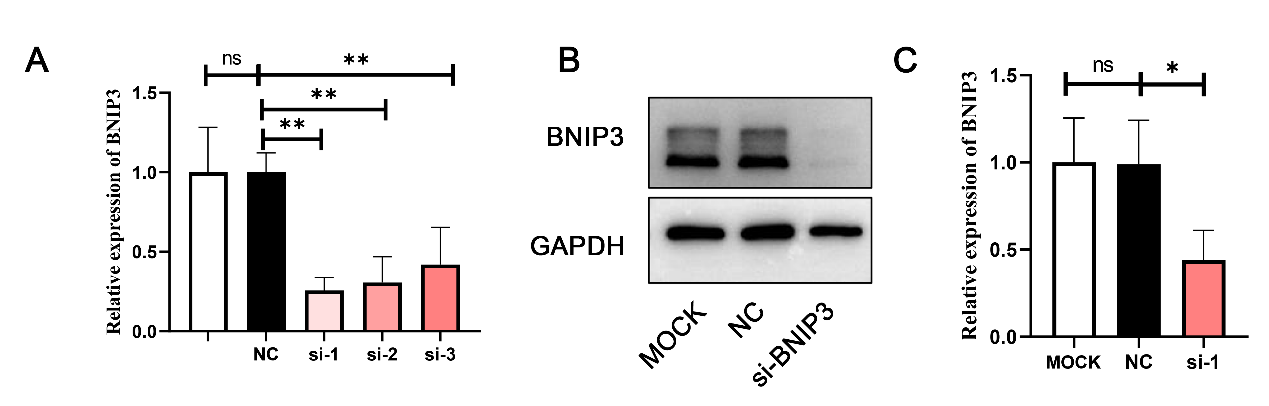


**Supplementary Figure 3. Verification of the knockdown efficiency of BNIP3-siRNAs.**

**Supplementary figure 4**


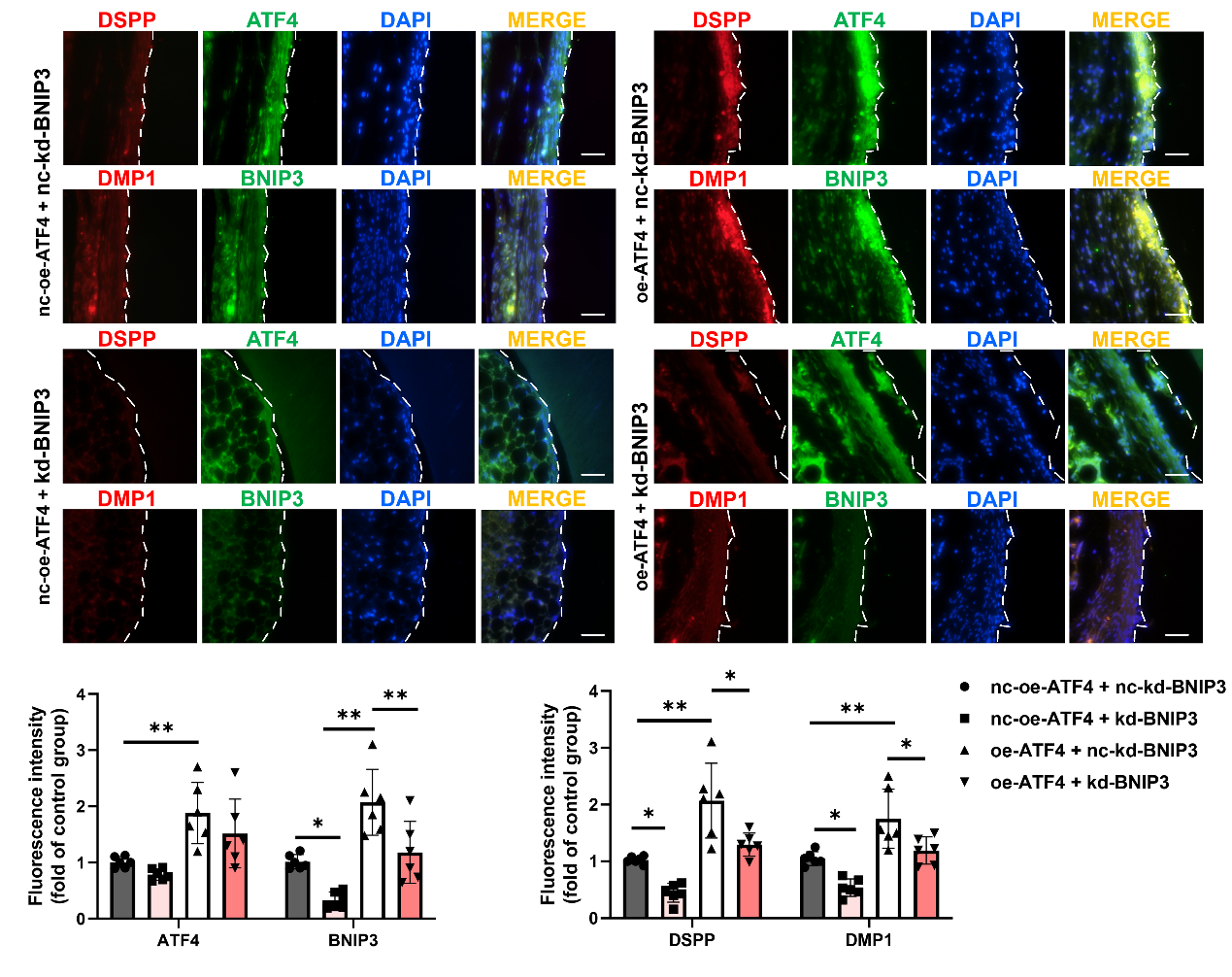


**Supplementary Figure 4. IF staining was used to assess the role of ATF4/BNIP3 in DPSC differentiation into odontoblasts in vivo, and the semiquantitative intensities of the areas with positive staining for ATF4, BNIP3, DMP1, and DSPP were measured**.

**Supplementary figure 5**


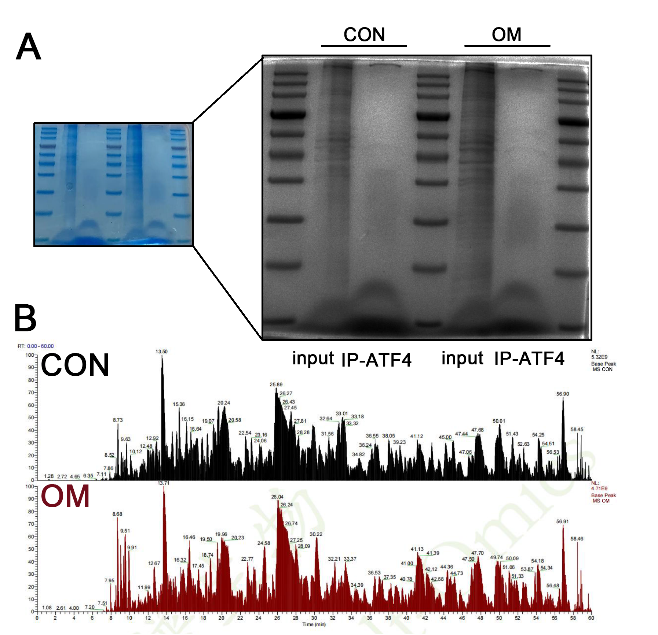


**Supplementary Figure 5. Proteins that interact with ATF4 in DPSCs differentiate into odontoblasts.**

A. The gel was stained with Coomassie blue to visualize the total proteins bound to ATF4 in the control and OM medium.

B. IP-MS results identifying ATF4-specific interacting proteins.

**Supplementary figure 6**


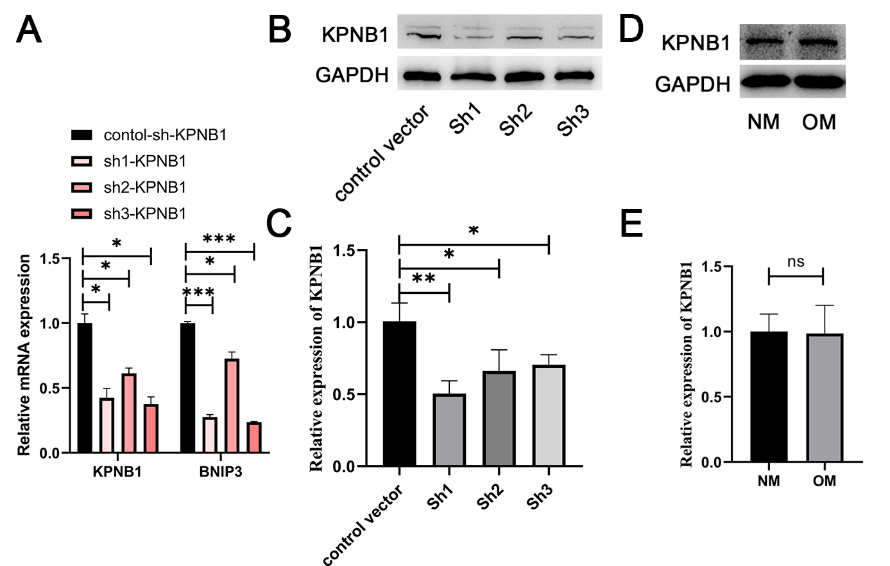


**Supplementary Figure 6. Examination of the transfection efficiency of sh-KPNB1.**

A. Verification of the most efficient KPNB1 knockdown sequence in transfection experiments.

B-C. Protein levels were determined by western blot analysis and normalized to that of GAPDH in terms of the relative intensity.

D-E. KPNB1 expression in DPSCs treated with CON or OM for 3 days was determined by western blot analysis and normalized to that of GAPDH in terms of the relative intensity.

**Supplementary figure 7**


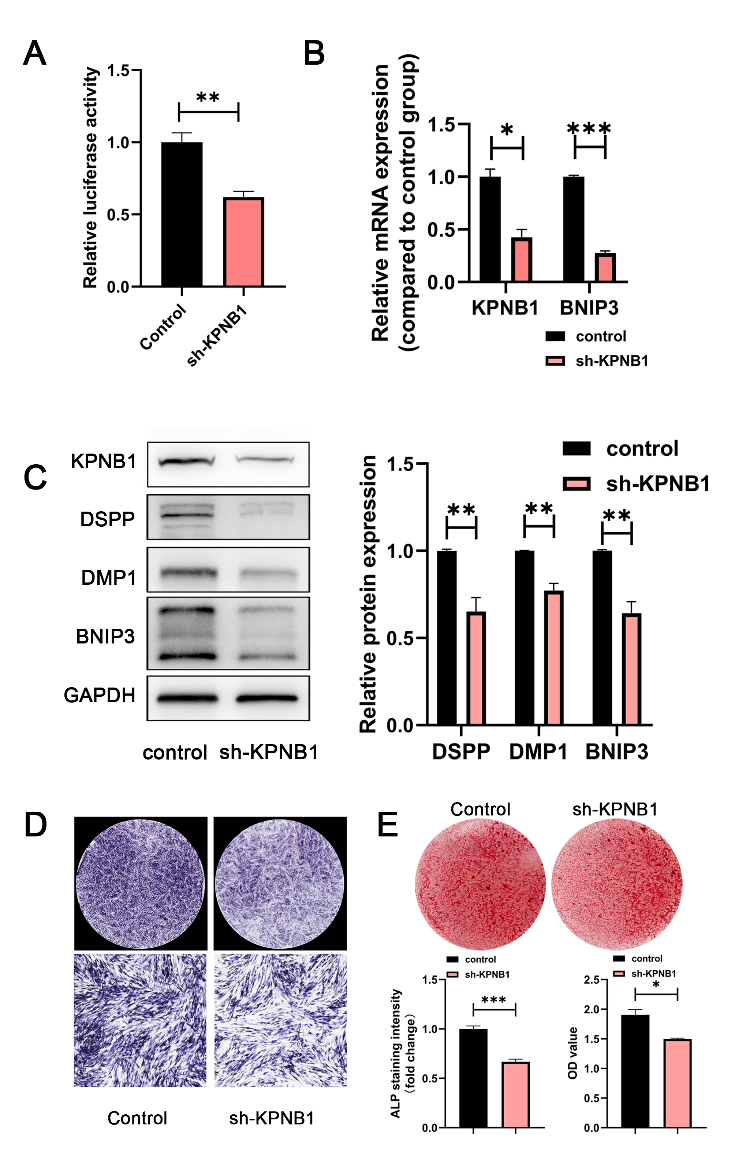


**Supplementary Figure 7. KPNB1 knockdown impaired both BNIP3 expression and DPSC differentiation ability.**

A. Dual‒luciferase reporter experiments were performed to assess BNIP3 promoter transcription.

B. RT‒qPCR analysis of KPNB1 and BNIP3 mRNA expression levels in KPNB1-knockdown DPSCs.

C. BNIP3 and odontoblastic marker expression levels were detected via western blotting (7 days) in DPSCs transfected with the control or sh-KPNB1 vector. The right panel shows the quantitative representation of the western blot band intensities.

D. ALP staining (7 days) of the control/sh-KPNB1 vector-transfected cells. Scale bar=500 μm. The right panel shows the semi-quantification of ALP staining intensity.

E. ARS staining (21 days) with control/sh-KPNB1 vector. The right panel shows the quantification of stained calcium deposits at 562 nm in DPSCs.

The data are presented as the means ± S. D. N values from 3 to 8; **p* < 0.05 and ***p* < 0.01 indicate significant differences between the indicated columns.
